# Bio-Inspired Anisotropic On-Manifold Guidance for Bearing-Only UAV Target Localization Under No-Fly Zone Constraints

**DOI:** 10.3390/biomimetics11080521

**Published:** 2026-07-23

**Authors:** Zeyuan Li, Linzhe Chen, Haoqiang Liu, Kelin Lu

**Affiliations:** School of Automation, Southeast University, Nanjing 210096, China; lizeyuan@seu.edu.cn (Z.L.); clinz@seu.edu.cn (L.C.); 220252168@seu.edu.cn (H.L.)

**Keywords:** bio-inspiration, target localization, Fisher information matrix, no-fly zones, sensor bias estimation, on-manifold modulation

## Abstract

In constrained environments with no-fly zones (NFZs), bearing-only UAV target localization requires improving UAV-target relative geometry while avoiding NFZs. Inspired by the avoidance behavior observed in flying insects driven by perceptual stimuli, this paper proposes a bio-inspired anisotropic on-manifold guidance method for bearing-only UAV target localization under NFZ constraints. First, to quantify the measurement information with respect to the UAV-target relative geometry under unknown sensor bias, a projected uncertainty field is proposed. Its spatial gradient drives a perception-guided control law, and its components are further used to characterize local information sensitivity as a perceptual stimulus. Second, this sensitivity is incorporated into NFZ margin adaptation to construct an anisotropic dual-layer manifold field, enabling the UAV to adapt the avoidance margin according to the local information distribution. An on-manifold modulation mechanism is then applied to generate feasible motion under NFZ constraints. Simulations in NFZ-free, nonconvex NFZ, and dense NFZ scenarios show that the proposed method generates UAV trajectories that avoid NFZs while maintaining accurate and convergent target localization.

## 1. Introduction

Unmanned aerial vehicles (UAVs), characterized by high maneuverability [1], low deployment cost [2], and mission versatility [3], have been widely used in reconnaissance and surveillance [4], search and rescue [5], remote sensing [6] and environmental monitoring [7]. If the UAV remains in a poor observation geometry for a prolonged period, system observability can be significantly weakened, which may cause the state estimation error to accumulate and degrade localization performance [8,9,10,11,12]. Moreover, passive sensors carried by lightweight UAVs may be affected by systematic bearing bias caused by installation misalignment and boresight calibration errors [13,14]. If the bias is ignored during localization, the target localization accuracy can be further degraded [15]. These two factors jointly constrain the achievable localization accuracy in bearing-only UAV systems.

For bearing-only target localization, existing studies have shown that observation geometry has a critical influence on localization performance. Fogel and Gavish analyzed observability from bearing measurements for targets governed by Nth-order dynamics, showing that the lack of sufficiently rich relative motion between the observer and the target can significantly degrade state identifiability [8]. Oshman and Davidson formulated trajectory design as the maximization of the determinant of the Fisher information matrix (FIM) under constraints, providing an early framework for trajectory optimization in bearing-only localization [16]. He et al. analyzed observability from a geometric perspective for two-dimensional bearing-only target localization. Based on this analysis, they derived an optimal single-step maneuver under heading constraints [17]. Li et al. further designed an observability-enhanced helical tracking trajectory in three-dimensional scenarios, demonstrating that persistent relative tangential motion is essential for improving estimation accuracy [18]. For bearing-only localization with sensor bias, Yang et al. jointly modeled the target position and a constant bearing bias and performed trajectory optimization using the determinant of the FIM [19]. Peng et al. drew on plant phototropism and incorporated the condition number of the observability matrix into control barrier function (CBF) constraints to maintain favorable observability during UAV trajectory planning for biased bearing localization [20]. Wang et al. proposed a method inspired by compound eye vision that constructs a maximum mean discrepancy based distributional observability metric for UAV trajectory optimization in bearing-only localization with sensor bias estimation [21].

However, the methods above do not account for no-fly zone (NFZ) constraints during observation geometry optimization [17,19,20,22]. For bearing-only localization, favorable estimation generally requires sufficient line of sight (LOS) variation induced by relative motion between the UAV and the target [23,24]. Relative tangential motion is a common and effective way to create such bearing variation [9,18,25]. When NFZs are present in the environment [26,27], the maneuvers required for observability improvement may conflict with the need to avoid restricted regions. As a result, avoidance corrections may compromise motion trends that would otherwise improve the UAV-target relative geometry. This issue is particularly pronounced near nonconvex, connected obstacles or narrow passages, where stagnation may arise due to undesired local minima [28,29,30,31].

To generate feasible motion under obstacle avoidance, several methods have been developed to construct real-time feasible motion fields [31,32]. Khansari-Zadeh and Billard developed a representative dynamical system modulation approach for real-time obstacle avoidance. Its core idea is to apply a local modulation matrix to the velocity field while preserving the attractor structure of the nominal dynamical system [28]. Subsequently, Huber and Billard proposed a closed-form obstacle avoidance method based on contraction theory, extending the modulation framework to moving convex obstacles and star-shaped nonconvex obstacles, and guaranteeing impenetrability of the obstacle boundary and asymptotic stability of the target point through contraction analysis [29]. To address local circumnavigation difficulties around nonconvex obstacles, Huber et al. developed a method that rotates the nominal nonlinear dynamics to improve motion generation near partially occluded workspaces and nonconvex obstacles [33]. More recently, Fourie et al. proposed an on-manifold modulation method, in which obstacle boundaries are modeled as navigable manifolds, enabling continuous circumnavigation and high-frequency real-time avoidance of general nonconvex obstacles [30]. However, such modulation frameworks are commonly applied to generic target-attracting nominal fields, and do not explicitly account for the relative-geometry requirements of bearing-only localization, where the nominal field should promote relative motion rather than pure target attraction.

Small flying insects exhibit obstacle avoidance behavior in cluttered environments using local perceptual cues, providing inspiration for UAV guidance in constrained settings. Previous studies have indicated that flying insects can use perceptual cues, such as optic flow, retinal expansion rate, and visual motion speed, to perform navigation, heading regulation, landing, and obstacle avoidance [34,35,36]. For example, blowflies adjust flight speed, meander amplitude, and saccadic turns according to arena width and obstacle layout, with these changes being partly associated with optic flow information [37]. Bumblebees also adjust their evasion response during obstacle flight, with evasion acceleration increasing with the relative retinal expansion velocity of approaching obstacles [38]. Moreover, obstacle avoidance in predatory robber flies is affected by the expansion rate of approaching obstacles in the visual field, producing steering deviations away from obstacles [39]. Inspired by the stimulus-induced directional deviations observed in the avoidance behavior of small flying insects, this paper introduces an anisotropic safety margin near NFZ boundaries. Local information sensitivity is used as a task-specific signal to modulate this margin according to the localization objective.

The main contributions of this paper are as follows:

(1) A perception-guided control law based on an instantaneous pseudo-observation is proposed. To establish a surrogate stimulus for the subsequent bio-inspired boundary adaptation, this paper characterizes the spatial distribution of measurement information as a field representation. Specifically, an instantaneous pseudo-observation is introduced at the current UAV position, from which an information-shaped projected uncertainty field is constructed over the UAV position space. The gradient-ascent direction of this field is used to construct the perception-guided control law, while its gradient components are further formulated as local information sensitivity.
(2) An anisotropic shifted boundary construction is proposed by incorporating local information sensitivity into NFZ margin adaptation, motivated by the directional avoidance tendency observed in flying insects under local perceptual stimuli. Directions with higher sensitivity are assigned smaller added margins to preserve informative motion, whereas directions with lower sensitivity are assigned larger added margins to promote boundary circumnavigation and preserve tangential motion. These local shifted boundaries are then fused into a dual-layer manifold field composed of an inner safety manifold and an outer guidance manifold.
(3) A modulation matrix and a branch selection mechanism are designed on the proposed dual-layer manifold field. This paper adopts a sample-based on-manifold modulation strategy to reshape the perception-guided vector near NFZ boundaries and generate feasible motion. To mitigate trajectory oscillations caused by repeated switching of the perception-guided modulation direction, a branch selection strategy is further introduced to enhance circumnavigation stability in complex constrained environments.

The remainder of this paper is organized as follows. Section 2 presents the problem statement, system model, and basic assumptions. Section 3 constructs the projected uncertainty field and develops the corresponding perception-guided control law. Section 4 formulates the local information sensitivity, and incorporates it into NFZ margin adaptation to construct an anisotropic dual-layer manifold field. Section 5 evaluates the proposed method through simulations in free-space and complex NFZ environments. Finally, Section 6 concludes this paper and discusses future work.

## 2. Problem Statement

### 2.1. System Modeling

**Figure 1:**
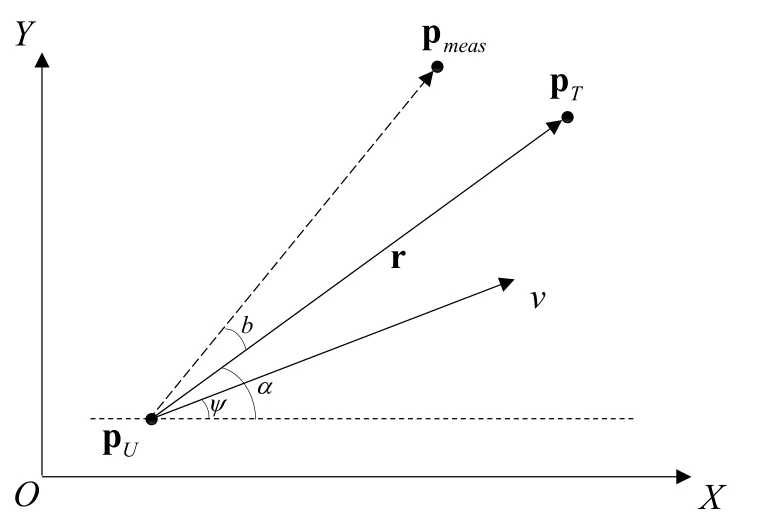
Bearing-only localization geometry with bias.

The geometric relationship between the UAV and the target considered in this paper is illustrated in Figure 1. A bearing-only localization problem for a stationary target in a two-dimensional plane is considered [19,20,21]. The UAV operates at a constant or regulated altitude, and each NFZ is modeled by its horizontal footprint. Let O−XY denote the global coordinate frame. Throughout this paper, $x\in R^{2}$ denotes a point in the workspace. The UAV position is denoted by $p_{U}\left(t\right)={\left[x_{U}\left(t\right),\;y_{U}\left(t\right)\right]}^{T}$, and the target position is denoted by $p_{T}\left(t\right)={\left[x_{T}\left(t\right),\;y_{T}\left(t\right)\right]}^{T}$. The angle α denotes the true bearing angle of the target with respect to the UAV, *b* denotes the bearing measurement bias of the sensor, ψ(t) is the heading angle of the UAV, *v* is the velocity of the UAV, and $p_{\mathrm{meas}}\left(t\right)$ denotes the measured target position corresponding to the biased bearing measurement. The relative position from the UAV to the target is denoted by $r\left(t\right)=p_{T}\left(t\right)-p_{U}\left(t\right)$.

The planar motion of the UAV is represented by the following kinematics:

(1) $$\left\{\begin{array}{cc} {\dot{p}}_{U}\left(t\right) & =g(\psi (t))v(t),\\ \dot{\psi }\left(t\right) & =\omega (t) \end{array}\right.$$

where $p_{U}\left(t\right)={\left[x_{U}\left(t\right),y_{U}\left(t\right)\right]}^{T}$, $g\left(\psi \right)={\left[\cos\psi ,\sin\psi \right]}^{T}$, and ω(t) is the heading rate.

At sampling instant *k*, the augmented target state is defined as [19]

(2) $$X_{T}\left(k\right)={\left[\begin{array}{ccc} x_{T}\left(k\right) & y_{T}\left(k\right) & b(k) \end{array}\right]}^{T}$$

where ${\left[x_{T}\left(k\right),\;y_{T}\left(k\right)\right]}^{T}$ is the target position, and b(k) is the sensor bias.

For the stationary target scenario considered in this paper, both the target position and the bias are treated as constant states. Small process noise is retained in the state model to account for modeling uncertainty and to maintain a well-conditioned covariance update:

(3) $$X_{T}\left(k+1\right)=\Phi_{k}\;X_{T}\left(k\right)+w\left(k\right)$$

where $\Phi_{k}=I_{3}$ is the state transition matrix, $I_{3}$ denotes the 3×3 identity matrix, and $w\left(k\right)∼N\left(0,Q_{k}\right)$ is the process noise. The relative position vector at sampling instant *k* is written as

$$r\left(k\right)=p_{T}\left(k\right)-p_{U}\left(k\right)={\left[r_{x}\left(k\right),\;r_{y}\left(k\right)\right]}^{T}.$$

Accordingly, the biased bearing-only measurement model is given by

(4) $$z\left(k\right)=\arctan\;\left(r_{y}\left(k\right),\;r_{x}\left(k\right)\right)+b\left(k\right)+\nu \left(k\right)$$

where $\nu \left(k\right)∼N\left(0,R_{k}\right)$ is the measurement noise. An extended Kalman filter (EKF) based on the above augmented state model is used to jointly estimate the target position and the sensor bias.

### 2.2. NFZ Representation and Modulation Framework

The NFZs in the workspace are regarded as obstacle regions, denoted by ${\left\{O_{m}\right\}}_{m=1}^{N_{o}}$. The corresponding feasible domain is defined as

(5) $$F≜R^{2}\setminus \overset{N_{o}}{\underset{m=1}{⋃}}O_{m}.$$

**Remark** **1.** *The obstacles considered in this paper are NFZs, which are modeled as restricted regions imposing geometric exclusion constraints on the UAV motion [26]. They constrain the feasible motion space but are not assumed to occlude the line of sight of the bearing sensor. Thus, the environmental influence is limited to trajectory feasibility, so that the analysis can focus on the effect of NFZ constraints on observation geometry evolution during maneuvering.*

For each obstacle region $O_{m}$, a continuous boundary function $\Gamma_{m}\left(x\right):R^{2}\to R$ is defined as

(6) $$\Gamma_{m}\left(x\right)=\left\{\begin{array}{cc} <1, & x\in O_{m},\\ =1, & x\in \partial O_{m},\\ >1, & x\in F \end{array}\right.$$

where the gradient $\nabla \Gamma_{m}$ points along the outward normal direction of the obstacle boundary. At time *t*, the boundary function used in this paper is required to be twice continuously differentiable ($C^{2}$ differentiable) with respect to the spatial variable *x* [29], namely

(7) $$\Gamma \left(x,t\right)\in C_{x}^{2}\left(R^{2}\right).$$

**Definition** **1** (γ-manifold)**.** *Following the γ-manifold terminology in [30], for a $C^{2}$ scalar boundary function $\Gamma \left(x,t\right):R^{2}\to R$, the γ-manifold is the one-dimensional manifold $M_{\gamma }\left(t\right)$ representing the level set Γ(x,t)=γ, with $\gamma \in R_{\geq 1}$.*

To ensure that the normal and tangential directions are well defined, the adopted γ-manifold is required to be regular:

(8) $$\nabla_{x}\Gamma \left(x,t\right)\neq 0,\;\forall \;x\in M_{\gamma }\left(t\right).$$

On this basis, the UAV motion is formulated as a locally modulated nominal dynamical system vector field:

(9) $${\dot{p}}_{U}=M\left(x\right)\;u_{g}\left(x\right)$$

where $M\left(x\right)\in R^{2\times 2}$ is the modulation matrix. In the neighborhood of obstacle boundaries, M(x) locally modulates the nominal vector field $u_{g}\left(x\right)$ to generate safe circumnavigation behavior [28]. The modulation matrix is written as

(10) $$M\left(x\right)=H\left(x\right)\Lambda \left(x\right)H^{T}\left(x\right)$$

where

(11) $$H\left(x\right)=\left[\begin{array}{cc} n(x) & t(x) \end{array}\right],\;\Lambda \left(x\right)=\left[\begin{array}{cc} 1-\frac{1}{\Gamma (x)} & 0\\ 0 & 1+\frac{1}{\Gamma (x)} \end{array}\right].$$

Near an obstacle boundary, the system suppresses the normal component and amplifies the tangential component of the nominal vector field, thereby locally reshaping the reference dynamics for geometric obstacle avoidance. The specific constructions of $u_{g}\left(x\right)$ and M(x) are presented in the following sections.

## 3. Perception-Guided Control Law

In biological navigation, flying insects extract local perceptual stimuli from their immediate sensory environment to regulate motion responses. Drawing on this principle, this paper abstracts the local distribution of measurement information at the current UAV position as a surrogate perceptual stimulus for the bearing-only localization task, and constructs a projected uncertainty field to quantify this stimulus. The spatial gradient of this field is used to construct a perception-guided control law.

Based on the augmented target state and the bearing measurement model with bias defined in Equations (2) and (4), the row Jacobian with respect to the augmented state $X_{T}\left(k\right)={\left[x_{T}\left(k\right),y_{T}\left(k\right),b\left(k\right)\right]}^{T}$ is

(12) $$h_{k}=\frac{\partial z(k)}{\partial X_{T}\left(k\right)}=\left[\begin{array}{ccc} -\frac{r_{y}\left(k\right)}{{∥r\left(k\right)∥}^{2}} & \frac{r_{x}\left(k\right)}{{∥r\left(k\right)∥}^{2}} & 1 \end{array}\right]=\left[\begin{array}{ccc} -\frac{\sin\alpha (k)}{∥r(k)∥} & \frac{\cos\alpha (k)}{∥r(k)∥} & 1 \end{array}\right]\in R^{1\times 3}.$$

For the augmented stationary target model in Equation (3), the state transition matrix is $\Phi_{k}=I_{3}$. Following the observability Gramian analysis for target localization with bearing bias [19], the local observability Gramian over a finite measurement window can be written as

(13) $$N_{k:k+N}=\sum_{i=k}^{k+N}\Phi_{i,k}^{T}h_{i}^{T}R_{i}^{-1}h_{i}\Phi_{i,k}=\sum_{i=k}^{k+N}h_{i}^{T}R_{i}^{-1}h_{i}.$$

This Gramian is a weighted sum of the outer products of the measurement Jacobians. Since $R_{i}≻0$, it can also be written as $N_{k:k+N}=O_{k:k+N}^{T}WO_{k:k+N}$, where $W=\mathrm{blkdiag}(R_{k}^{-1},\ldots ,R_{k+N}^{-1})≻0$ and $O_{k:k+N}={\left[h_{k}^{T},\ldots ,h_{k+N}^{T}\right]}^{T}$. Therefore, $N_{k:k+N}$ and $O_{k:k+N}$ have the same rank. A necessary local observability condition of the linearized system for jointly estimating the target position and the bearing bias is

(14) $$\mathrm{rank}\left(N_{k:k+N}\right)=3.$$

This condition requires sufficiently diverse relative geometries between the UAV and the target. In particular, for target localization with bearing bias, at least three bearing measurements with proper observer geometry are required [19]. Otherwise, the Gramian becomes singular, and the target position and bearing bias cannot be jointly identified. Static observation or motion with an almost unchanged relative bearing angle leads to linearly dependent or ill-conditioned rows in $O_{k:k+N}$, resulting in an unobservable or weakly observable local linearized system [17].

The rank condition above provides a qualitative local observability criterion, but does not quantify the degree of observability. For nonlinear state estimation problems, the Cramér–Rao lower bound (CRLB) provides a theoretical lower bound on the error covariance of any unbiased estimator [40]. Let $J_{k}$ denote the FIM at time step *k*. Then, the filtering error covariance satisfies $P_{k}⪰J_{k}^{-1}$. Maximizing the determinant of $J_{k}$ minimizes the volume of the uncertainty ellipsoid defined by $J_{k}^{-1}$, thereby improving the target localization accuracy. For the scalar bearing measurement, its recursive information form is given by [41]

(15) $$\begin{array}{cc} J_{k+1}= & Q_{k}^{-1}+h_{k+1}^{T}R_{k+1}^{-1}h_{k+1}-Q_{k}^{-1}\Phi_{k}{\left(J_{k}+\Phi_{k}^{T}Q_{k}^{-1}\Phi_{k}\right)}^{-1}\Phi_{k}^{T}Q_{k}^{-1} \end{array}$$

where $h_{k+1}$ is the measurement Jacobian evaluated at the current EKF linearization point. The information matrix is initialized as $J_{0}=P_{0}^{-1}$ and subsequently propagated by Equation (15).

To construct a reactive guidance metric that is computable at each sampling instant, an instantaneous pseudo-observation method is introduced. The pseudo-observation is evaluated at the current UAV observation position as an instantaneous measurement update, with no process-noise propagation involved, and is not applied to the filter. The corresponding instantaneous information update is defined as

(16) $${\tilde{J}}_{k}=J_{k}+\frac{1}{\sigma_{\theta }^{2}}h_{k}^{T}h_{k}$$

where $\sigma_{\theta }^{2}$ denotes the variance of the bearing measurement noise.

According to the matrix determinant lemma, one obtains

(17) $$\det\;\left({\tilde{J}}_{k}\right)=\det\left(J_{k}\right)\left(1+\frac{1}{\sigma_{\theta }^{2}}h_{k}J_{k}^{-1}h_{k}^{T}\right).$$

Since both $\det(J_{k})$ and $\sigma_{\theta }^{2}$ are independent of the instantaneous observation position perturbation, the FIM determinant increment induced by this instantaneous pseudo-observation is a positive scalar multiple of the following scalar surrogate:

(18) $$V_{k}≜h_{k}J_{k}^{-1}h_{k}^{T}.$$

We refer to $V_{k}$ as an information-shaped projected uncertainty field. For a fixed current information state $J_{k}$, this scalar measures how the state uncertainty represented by $J_{k}^{-1}$ is projected onto the local measurement direction defined by $h_{k}$. Therefore, its spatial gradient guides the UAV toward observation positions where the bearing measurement direction $h_{k}$ aligns with the directions of highest state uncertainty under $J_{k}^{-1}$, thereby maximizing the expected information gain.

To explicitly separate the effects of range contraction and LOS rotation, this paper reparameterizes the observation geometry using relative polar coordinates (r,ϕ). The observation Jacobian can then be written as

(19) $$h\left(r,\phi \right)=\left[\begin{array}{ccc} -\sin\phi /r & \cos\phi /r & 1 \end{array}\right].$$

Although the bias state does not vary with the observation position, the cross-coupling between position and bias in $J_{k}^{-1}$ still causes $V_{k}$ to vary across the observation plane. Let

(20) $$W≜J_{k}^{-1}=\left[\begin{array}{ccc} W_{11} & W_{12} & W_{13}\\ W_{12} & W_{22} & W_{23}\\ W_{13} & W_{23} & W_{33} \end{array}\right].$$

Substituting the above expression into Equation (18) yields the following closed-form expression with respect to (r,ϕ):

(21) $$V\left(r,\phi \right)=\frac{Q_{pos}\left(\phi \right)}{r^{2}}+\frac{2\;Q_{cross}\left(\phi \right)}{r}+W_{33}$$

where

(22) $$Q_{pos}\left(\phi \right)≜W_{11}\sin^{2}\;\phi -2W_{12}\sin\phi \cos\phi +W_{22}\cos^{2}\;\phi ,$$

(23) $$Q_{cross}\left(\phi \right)≜W_{23}\cos\phi -W_{13}\sin\phi$$

with $Q_{pos}\left(\phi \right)$ quantifying the contribution of the position subblock of $J_{k}^{-1}$ when projected onto the direction normal to the current LOS, whereas $Q_{cross}\left(\phi \right)$ captures the additional contribution caused by the coupling between position and bias. Taking derivatives of Equation (21) with respect to *r* and ϕ, with the tangential component scaled by 1/r, the radial and tangential gradient components are obtained as

(24) $$g_{r}=\frac{\partial V}{\partial r}=-\frac{2\;Q_{pos}\left(\phi \right)}{r^{3}}-\frac{2\;Q_{cross}\left(\phi \right)}{r^{2}},$$

(25) $$g_{\phi }=\frac{1}{r}\frac{\partial V}{\partial \phi }=\frac{Q_{pos}^{\prime }\left(\phi \right)}{r^{3}}+\frac{2\;Q_{cross}^{\prime }\left(\phi \right)}{r^{2}}.$$

The quantities $g_{r}$ and $g_{\phi }$ are the radial and tangential components of the gradient of $V_{k}$, respectively, in the polar basis defined by the current LOS direction. The term $Q_{pos}\left(\phi \right)$ can be written as a quadratic form

(26) $$Q_{pos}\left(\phi \right)=e_{\phi }^{T}\left(\phi \right)W_{pos}e_{\phi },\;e_{\phi }={\left(-\sin\phi ,\cos\phi \right)}^{T}$$

where

(27) $$W_{pos}=\left[\begin{array}{cc} W_{11} & W_{12}\\ W_{12} & W_{22} \end{array}\right].$$

Since $J_{k}≻0$, one has $W=J_{k}^{-1}≻0$, and thus $W_{pos}≻0$. Therefore, $Q_{pos}\left(\phi \right)>0$ holds for any ϕ. Furthermore,

(28) $$Q_{pos}^{\prime }\left(\phi \right)=\left(W_{11}-W_{22}\right)\sin2\phi -2W_{12}\cos2\phi =A\sin\left(2\phi -\delta \right)$$

where

(29) $$A=\sqrt{{\left(W_{11}-W_{22}\right)}^{2}+4W_{12}^{2}},\;\delta =\arctan\left(2W_{12},\;W_{11}-W_{22}\right).$$

The following discussion focuses on the nondegenerate case A>0. In the bias-free case, $W_{pos}$ becomes isotropic when A=0, the tangential baseline drive degenerates, and the field is dominated by radial contraction.

Next, perform the spectral decomposition

(30) $$W_{pos}=U\left[\begin{array}{cc} \lambda_{\max} & 0\\ 0 & \lambda_{\min} \end{array}\right]U^{T},\;\lambda_{\max}\geq \lambda_{\min}>0.$$

According to the extremal property of the Rayleigh quotient, $Q_{pos}\left(\phi \right)$ reaches its maximum value $\lambda_{\max}$ when $e_{\phi }$ is aligned with the eigenvector corresponding to the largest eigenvalue of $W_{pos}$. Therefore, in the bias-free case, the role of $g_{\phi }$ can be interpreted as driving the LOS bearing toward the principal observation geometry favored by the position subblock. Equivalently, the LOS direction tends to align with the eigenvector corresponding to the smallest eigenvalue. Together with the radial attraction represented by $g_{r}$, this geometric effect can locally induce a spiral-like approach behavior that combines inward motion with angular correction. When the bias coupling is further taken into account, the local gradient direction can be shifted according to the coupling terms in W.

Let the cross-term vector be $c_{b}≜{\left[W_{13},\;W_{23}\right]}^{T}$, and define the local LOS and tangential bases as

$$e_{r}={\left[\cos\phi ,\;\sin\phi \right]}^{T},\;e_{\phi }={\left[-\sin\phi ,\;\cos\phi \right]}^{T}.$$

Then the bias-coupling term and its angular derivative can be compactly written as

$$Q_{\mathrm{cross}}\left(\phi \right)=c_{b}^{T}e_{\phi },\;Q_{\mathrm{cross}}^{\prime }\left(\phi \right)=-c_{b}^{T}e_{r}.$$

Therefore,

(31) $$g_{r}=-\frac{2}{r^{3}}Q_{\mathrm{pos}}\left(\phi \right)-\frac{2}{r^{2}}c_{b}^{T}e_{\phi },\;g_{\phi }=\frac{1}{r^{3}}Q_{\mathrm{pos}}^{\prime }\left(\phi \right)-\frac{2}{r^{2}}c_{b}^{T}e_{r}.$$

When the coupling between position and bias is included, the projections of $c_{b}$ onto the local tangential and radial directions introduce signed corrections to the gradient components. Specifically, $c_{b}^{T}e_{\phi }$ affects the radial component $g_{r}$, whereas $c_{b}^{T}e_{r}$ affects the tangential component $g_{\phi }$. The sign and magnitude of these corrections depend on the current LOS geometry and on the uncertainty structure represented by $J_{k}^{-1}$. Therefore, the coupling effect cannot be assigned a fixed global direction; instead, it modifies the information gradient locally along the trajectory.

**Figure 2:**
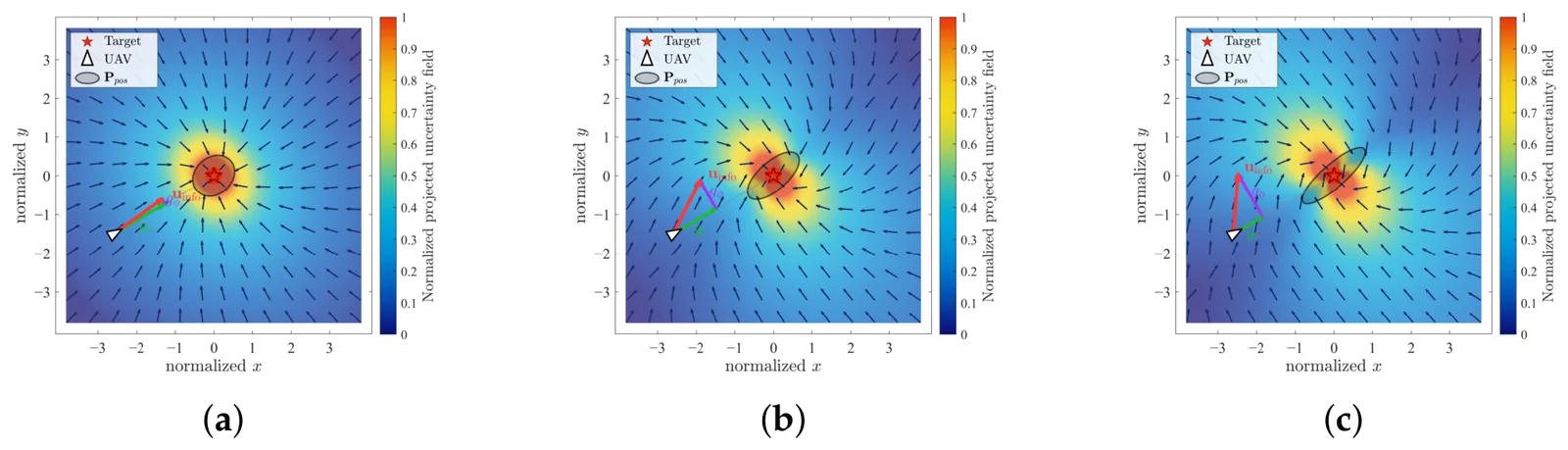
Normalized projected uncertainty field and perception-guided vector under three representative position covariance shapes: (**a**) near-isotropic, (**b**) moderate anisotropy, and (**c**) strong anisotropy. The green, purple, and red arrows denote the radial contribution $g_{r}$, the tangential contribution $g_{\phi }$, and the synthesized perception-guided vector $u_{\mathrm{info}}$, respectively.

Figure 2 illustrates the normalized projected uncertainty field and the decomposed guidance vector under three representative shapes of the position covariance $P_{pos}$. In bearing-only localization, a single bearing measurement does not directly provide range information along the LOS direction. As a result, when sufficient LOS variation is absent, the position uncertainty tends to be larger along the LOS direction. When $P_{pos}$ is nearly isotropic, the gradient of $V_{k}$ is mainly governed by the radial component $g_{r}$, and the resulting guidance vector is biased toward radial approach. As the eccentricity of $P_{pos}$ increases along the LOS direction, the tangential component $g_{\phi }$ becomes more pronounced, and the guidance direction gradually shifts toward a compound maneuver that combines radial approach with lateral motion.

Because the gradient of $V_{k}$ is expressed in the target-relative frame whereas the UAV moves in the global position frame, a sign reversal is applied, and the raw guidance vector in free space is written as

(32) $$u_{\mathrm{info}}^{\prime }\left(t\right)=-R\left(\hat{\phi }\right)\left[\begin{array}{c} \partial V/\partial \hat{r}\\ {\hat{r}}^{-1}\;\partial V/\partial \hat{\phi } \end{array}\right],\;R\left(\hat{\phi }\right)=\left[\begin{array}{cc} \cos\hat{\phi } & -\sin\hat{\phi }\\ \sin\hat{\phi } & \cos\hat{\phi } \end{array}\right]$$

where $\hat{r}$ and $\hat{\phi }$ are the estimated range and bearing angle with respect to the target estimate.

The normalized perception-guided direction used for subsequent modulation is defined as

(33) $${\bar{u}}_{\mathrm{info}}\left(t\right)=\frac{u_{\mathrm{info}}^{\prime }\left(t\right)}{∥u_{\mathrm{info}}^{\prime }\left(t\right)∥+\varepsilon_{u}},\;\varepsilon_{u}>0.$$

To avoid excessive radial convergence near the estimated target, a local standoff guidance field is introduced in the transition region. Let $g_{\phi }^{\mathrm{in}}$ denote the value of $g_{\phi }$ evaluated at the instant the UAV enters the region $\hat{r}\leq R_{\mathrm{safe}}+R_{w}$, and held fixed during the current standoff phase. The standoff guidance field is defined as

(34) $${\bar{u}}_{\mathrm{st}}\left(t\right)=\frac{k_{r}\left(\hat{r}-R_{\mathrm{safe}}\right)e_{r}-\mathrm{sgn}\left(g_{\phi }^{\mathrm{in}}\right)e_{\phi }}{∥k_{r}\left(\hat{r}-R_{\mathrm{safe}}\right)e_{r}-\mathrm{sgn}\left(g_{\phi }^{\mathrm{in}}\right)e_{\phi }∥+\varepsilon_{u}}.$$

The sign of $g_{\phi }^{\mathrm{in}}$ is kept fixed during the standoff maneuver. The blending weight is defined by

(35) $$s_{R}\left(\hat{r}\right)={\mathrm{sat}}_{\left[0,1\right]}\left(\frac{R_{\mathrm{safe}}+R_{w}-\hat{r}}{R_{w}}\right),\;\sigma_{R}\left(\hat{r}\right)=3s_{R}^{2}-2s_{R}^{3}$$

where ${\mathrm{sat}}_{\left[0,1\right]}\left(x\right)=\min\left\{1,\max\left\{0,x\right\}\right\}$.

The final guidance vector is given by

(36) $$u_{\mathrm{info}}\left(t\right)=\left(1-\sigma_{R}\left(\hat{r}\right)\right){\bar{u}}_{\mathrm{info}}\left(t\right)+\sigma_{R}\left(\hat{r}\right){\bar{u}}_{\mathrm{st}}\left(t\right).$$

Let $t_{k}=k\Delta T$, k=0,1,2,…, be the sampling instants. At each $t_{k}$, the gradient $\nabla_{p}V_{k}$ is evaluated from the current state estimate and information state $J_{k}$, and held constant over $\left[t_{k},t_{k+1}\right)$ via zero-order hold. In the long-range phase $\hat{r}\geq R_{\mathrm{safe}}+R_{w}$, $u_{\mathrm{info}}$ reduces to ${\bar{u}}_{\mathrm{info}}$; within the transition band it is smoothly blended with the circular-orbiting command; for $\hat{r}\leq R_{\mathrm{safe}}$ it reduces to the circular-orbiting command alone.

For the UAV model in Equation (1), the perception-guided control law is then implemented by mapping $u_{\mathrm{info}}$ to speed and heading commands as

(37) $$\left\{\begin{array}{cc} \psi_{\mathrm{cmd}}\left(t\right) & =\arctan\left(u_{\mathrm{info},y}\left(t\right),u_{\mathrm{info},x}\left(t\right)\right),\\ e_{\psi }\left(t\right) & =\arctan\left(\sin\left(\psi_{\mathrm{cmd}}\left(t\right)-\psi \left(t\right)\right),\cos\left(\psi_{\mathrm{cmd}}\left(t\right)-\psi \left(t\right)\right)\right),\\ v(t) & =v_{\max}\max\left\{0,\cos e_{\psi }\left(t\right)\right\},\\ \omega (t) & ={\mathrm{sat}}_{\left[-\omega_{\max},\;\omega_{\max}\right]}\left(k_{\psi }e_{\psi }\left(t\right)\right). \end{array}\right.$$

The resulting control law is $u_{\mathrm{cmd}}\left(t\right)={\left[v\left(t\right),\omega \left(t\right)\right]}^{T}$.

## 4. Bio-Inspired Anisotropic On-Manifold Modulation

Inspired by the directional steering deviations observed in flying insects under asymmetric local stimuli, this section incorporates the local information sensitivity derived from the projected uncertainty field in Section 3 into NFZ margin adaptation to construct anisotropic shifted boundaries. A modulation matrix is then defined on the manifold generated by the proposed dual-layer field, so that the system can avoid NFZ boundaries while retaining the motion preferred by the perception-guided control law.

### 4.1. Anisotropic Shifted Boundary Construction

This subsection defines local scale parameters from the gradient of the projected uncertainty field and uses them to construct anisotropic shifted boundaries.

#### 4.1.1. Directional Information Sensitivity and Anisotropic Shifted Boundaries

Fabian et al. [39] interpreted the obstacle avoidance behavior of a small flying insect during target interception as a guidance law with a deviation term:

$${\dot{\psi }}_{\mathrm{bio}}=f_{\mathrm{base}}\left(\dot{\lambda }\right)+f_{\mathrm{dev}}\left(\dot{\vartheta }\right).$$

Here, $\dot{\lambda }$ denotes the bearing-rate cue associated with the basic guidance behavior, whereas $\dot{\vartheta }$ denotes the visual expansion rate of the obstacle. This form shows that the strength of a local stimulus can regulate the deviation of the avoidance response.

For the UAV localization task, the task-relevant stimulus is defined from the information carried by the bearing measurement z(k). Through the projected uncertainty field $V_{k}$, the local sensitivity *S* indicates how valuable the current geometry is for localization. This stimulus is therefore used to adjust the avoidance margin:

$$l\left(d\right)=l_{\mathrm{base}}+l_{\mathrm{dev}}\left(S\right).$$

Here, *d* denotes a local direction, $l_{\mathrm{base}}$ is the basic avoidance margin, and $l_{\mathrm{dev}}\left(S\right)$ is the deviation margin induced by the local information sensitivity.

Consider the *i*-th sampled reference point $c_{i}$ on the NFZ boundary. Let $g_{r}^{\left(i\right)}$ and $g_{\phi }^{\left(i\right)}$ denote the radial and tangential components of the gradient of the projected uncertainty field obtained in Section 3, respectively. The directional sensitivities with logarithmic compression are defined as

(38) $$S_{r}^{\left(i\right)}=\log\;\left(1+\kappa {\left[-g_{r}^{\left(i\right)}\right]}_{+}\right),\;S_{\phi }^{\left(i\right)}=\log\;\left(1+\kappa |g_{\phi }^{\left(i\right)}|\right)$$

where ${\left[z\right]}_{+}=\max\left(z,0\right)$, and κ>0 is a scaling coefficient. The rectified term ${\left[-g_{r}^{\left(i\right)}\right]}_{+}$ ensures that the radial sensitivity is activated only when moving inward along the radial direction locally increases $V_{k}$ (i.e., $g_{r}^{\left(i\right)}<0$), whereas it vanishes for $g_{r}^{\left(i\right)}\geq 0$ without introducing additional radial anisotropic expansion.

The local information sensitivity plays the role of the local perceptual stimulus, and determines how much margin should be assigned in different local directions. For an arbitrary local direction $d\in \{e_{r}^{\left(i\right)},e_{\phi }^{\left(i\right)}\}$, the normalized directional information sensitivity is defined as

(39) $$\omega_{I}^{\left(i\right)}\left(d\right)=\frac{\mu_{s}S_{I}^{\left(i\right)}\left(d\right)}{1+\mu_{s}S_{I}^{\left(i\right)}\left(d\right)}\in \left[0,1\right)$$

where $S_{I}^{\left(i\right)}\left(e_{r}^{\left(i\right)}\right)=S_{r}^{\left(i\right)}$, $S_{I}^{\left(i\right)}\left(e_{\phi }^{\left(i\right)}\right)=S_{\phi }^{\left(i\right)}$, and $\mu_{s}>0$ is a tuning coefficient.

The radial and tangential scale parameters at sample point $c_{i}$, expressed in the local LOS frame, are defined as

(40) $$l_{r}^{\left(i\right)}=\delta_{\min}+\eta_{t}\left(1-\omega_{I}^{\left(i\right)}\left(e_{r}^{\left(i\right)}\right)\right)=\delta_{\min}+\frac{\eta_{t}}{1+\mu_{s}S_{r}^{\left(i\right)}},$$

(41) $$l_{\phi }^{\left(i\right)}=\delta_{\min}+\eta_{t}\left(1-\omega_{I}^{\left(i\right)}\left(e_{\phi }^{\left(i\right)}\right)\right)=\delta_{\min}+\frac{\eta_{t}}{1+\mu_{s}S_{\phi }^{\left(i\right)}}$$

where $\delta_{\min}>0$ is the minimum geometric safety margin, and $\eta_{t}>0$ is a dynamic gain that varies with the estimation uncertainty. The above scales can be written in a unified form

(42) $$l^{\left(i\right)}\left(d\right)=\delta_{\min}+\eta_{t}\left(1-\omega_{I}^{\left(i\right)}\left(d\right)\right).$$

To avoid a fixed gain being either overly conservative or insufficiently protective across varying estimation uncertainty, $\eta_{t}$ is updated adaptively according to the estimation uncertainty, so that the overall margin scale contracts progressively as the localization accuracy improves. Let $P_{t}$ denote the current error covariance matrix, let $P_{pos,t}$ be its 2×2 position covariance subblock, and let $P_{pos,0}$ be the initial position covariance subblock. Define

(43) $$u_{t}=\min\;\left(1,\frac{\mathrm{tr}(P_{pos,t})}{\mathrm{tr}(P_{pos,0})}\right),\;\eta_{t}=\eta_{\min}+\left(\eta_{\max}-\eta_{\min}\right)u_{t}^{1/k_{c}}$$

where $\eta_{\min}$ and $\eta_{\max}$ are the lower and upper bounds of the dynamic gain, respectively, and $k_{c}>0$ is a shaping parameter. This design keeps the local scale relatively large when the uncertainty is high, and gradually returns the system to a tighter geometric constraint after the estimation converges.

Furthermore, $\omega_{I}^{\left(i\right)}\left(d\right)$ denotes the normalized information sensitivity along direction d, which plays the role of the perceptual stimulus in the bio-inspired margin adaptation. When the sensitivity along d is high, $\omega_{I}^{\left(i\right)}\left(d\right)$ increases, the added margin decreases, and the local boundary is tightened, which helps preserve compact informative motion along that direction. When the sensitivity along d is low, $\omega_{I}^{\left(i\right)}\left(d\right)$ decreases, the added margin increases, and the local boundary expands outward, which strengthens the tendency to take a wider detour around the NFZ so as to preserve relative motion that is beneficial for localization. From the viewpoint of obstacle avoidance, $\eta_{t}\left(1-\omega_{I}^{\left(i\right)}\left(d\right)\right)$ can be interpreted as a local avoidance deviation shaped by information sensitivity, analogous to the directional steering deviations observed in flying insects in response to local perceptual stimuli.

For a sample point $c_{i}$, this paper uses a local orthonormal basis aligned with the LOS from the sample point to the estimated target:

(44) $$R_{i}\left(t\right)=\left[\begin{array}{cc} e_{r}^{\left(i\right)}\left(t\right) & e_{\phi }^{\left(i\right)}\left(t\right) \end{array}\right],\;e_{r}^{\left(i\right)}\left(t\right)=\frac{{\hat{p}}_{T}\left(t\right)-c_{i}}{∥{\hat{p}}_{T}\left(t\right)-c_{i}∥},\;e_{\phi }^{\left(i\right)}\left(t\right)=R_{\pi /2}e_{r}^{\left(i\right)}\left(t\right)$$

where ${\hat{p}}_{T}\left(t\right)$ is the current target position estimate, and $e_{r}^{\left(i\right)}\left(t\right)$ and $e_{\phi }^{\left(i\right)}\left(t\right)$ denote the LOS radial direction and its tangential unit vector obtained by a counterclockwise rotation of π/2, respectively. Therefore, the principal axes of the anisotropic shifted ellipse are aligned with the instantaneous LOS frame.

The local anisotropic metric matrix induced by sample point $c_{i}$ at time *t* is written as

(45) $$A_{i}\left(t\right)=R_{i}\left(t\right)\left[\begin{array}{cc} 1/{\left(l_{r}^{\left(i\right)}\left(t\right)\right)}^{2} & 0\\ 0 & 1/{\left(l_{\phi }^{\left(i\right)}\left(t\right)\right)}^{2} \end{array}\right]R_{i}^{T}\left(t\right).$$

The corresponding anisotropic shifted boundary is represented by

(46) $$B_{i}\left(t\right)=\left\{x\;|\;{\left(x-c_{i}\right)}^{T}A_{i}\left(t\right)\left(x-c_{i}\right)=1\right\}.$$

Geometrically, $B_{i}\left(t\right)$ is the unit level set of an anisotropic quadratic metric defined by $A_{i}\left(t\right)$. Its shape is determined by the relative magnitudes of $l_{r}^{\left(i\right)}\left(t\right)$ and $l_{\phi }^{\left(i\right)}\left(t\right)$.

**Remark** **2** (Geometry of anisotropic shifted boundaries under directional information sensitivity)**.** *The scale parameters $l_{r}^{\left(i\right)}$ and $l_{\phi }^{\left(i\right)}$ monotonically decrease with the corresponding sensitivities, with $\delta_{\min}$ as the lower bound, and the shape of $B_{i}\left(t\right)$ is determined by the ratio $S_{r}^{\left(i\right)}/S_{\phi }^{\left(i\right)}$. When $S_{r}^{\left(i\right)}>S_{\phi }^{\left(i\right)}$, the boundary contracts along the LOS radial direction and expands along the LOS tangential direction. When $S_{r}^{\left(i\right)}<S_{\phi }^{\left(i\right)}$, the boundary contracts along the LOS tangential direction and expands along the LOS radial direction. When $S_{r}^{\left(i\right)}=S_{\phi }^{\left(i\right)}$, or when both sensitivities vanish, the boundary reduces to an isotropic circular boundary.*

**Figure 3:**
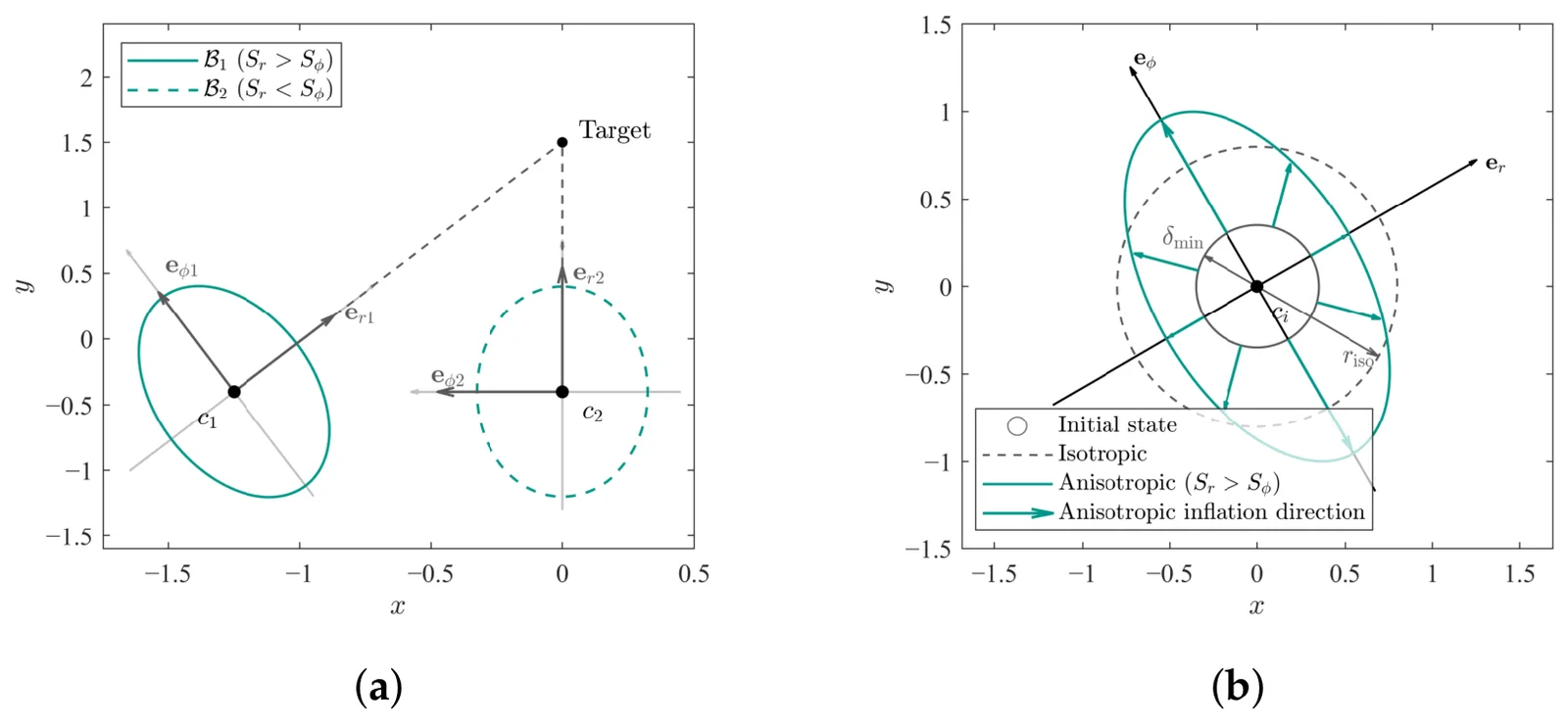
Illustration of the proposed anisotropic shifted boundary. (**a**) Two local boundary examples under different directional sensitivities, where $e_{r}$ points toward the target and $e_{\phi }$ denotes the counterclockwise tangential basis. (**b**) Comparison between the isotropic boundary and the anisotropic shifted boundary.

To illustrate this local geometric effect, Figure 3 shows the anisotropic shifted boundary induced by a single boundary sample point under the local anisotropic metric.

#### 4.1.2. Construction of the Dual-Layer Manifold Field

To combine the local anisotropic boundaries into a single field near the obstacle, this paper builds a smooth approximation of the nearest local boundary field from sampled boundary points [30]. The local quadratic forms are fused by inverse distance weighting, which yields a continuous approximate obstacle field for modulation outside the boundary and for basis construction. The Euclidean inverse distance weight is defined as

(47) $$\alpha_{i}\left(x\right)={\left(∥x-c_{i}{∥}^{n}+\varepsilon_{w}\right)}^{-1}$$

where $\varepsilon_{w}>0$ is a regularization term, and n≥2 is chosen as an even integer so that $\alpha_{i}\left(x\right)$ is $C^{2}$ with respect to *x*. Let $N_{s}$ denote the number of sampled NFZ boundary points. The raw time-varying anisotropic field is then defined as

(48) $${\tilde{\Gamma }}_{t}\left(x,t\right)=\frac{\sum_{i=1}^{N_{s}}\alpha_{i}\left(x\right){\left(x-c_{i}\right)}^{T}A_{i}\left(t\right)\left(x-c_{i}\right)}{\sum_{i=1}^{N_{s}}\alpha_{i}\left(x\right)}.$$

Equation (48) is essentially a smooth approximation of the nearest local boundary field. Its boundary shape and level-set topology depend on the boundary sampling density and on the weight exponent *n*. In implementation, obstacle boundaries are first sampled densely with a fixed spacing *h* between adjacent samples.

On this basis, the separation required between the UAV and the obstacle boundary is used to build the inner safety manifold. A fixed isotropic inner safety manifold with offset scale $\delta_{\min}$ is defined as

(49) $$\gamma_{l}\left(x\right)=\frac{\sum_{i=1}^{N_{s}}\alpha_{i}\left(x\right){∥x-c_{i}∥}^{2}/\delta_{\min}^{2}}{\sum_{i=1}^{N_{s}}\alpha_{i}\left(x\right)}.$$

Let

(50) $$\eta_{b}\left(x\right)=1-\frac{1}{\gamma_{l}\left(x\right)},\;\beta_{b}\left(x\right)=1-\exp\;\left(-\kappa_{\beta }\eta_{b}{\left(x\right)}^{2}\right),\;\kappa_{\beta }>0.$$

The outer guidance field that remains consistent with the boundary is then defined as

(51) $$\Gamma_{t}\left(x,t\right)=\left(1-\beta_{b}\left(x\right)\right)\gamma_{l}\left(x\right)+\beta_{b}\left(x\right){\tilde{\Gamma }}_{t}\left(x,t\right).$$

A larger $\kappa_{\beta }$ confines the transition between the inner and outer fields to a narrower neighborhood of $\gamma_{l}=1$, causing the anisotropic outer field to become dominant more rapidly away from the safety boundary.

In the following, it is assumed that the boundary samples are sufficiently dense, and that $\delta_{\min}$ and the weighting parameters are chosen so that $\gamma_{l}\left(x\right)=1$ forms a closed inner safety manifold enclosing the obstacle region. For a given parameter $\gamma_{\mathrm{ref}}>1$, the scalar field $\Gamma_{t}\left(x,t\right)$ defines an outer guidance manifold outside this safety manifold, and the level set $\Gamma_{t}\left(x,t\right)=\gamma_{\mathrm{ref}}$ is selected as the reference guidance layer. This paper adopts a dual-layer structure: the inner safety manifold $\gamma_{l}\left(x\right)=1$ encloses the obstacle region and defines the safety boundary used for the forward invariance guarantee, while the outer guidance manifold $\Gamma_{t}\left(x,t\right)=\gamma_{\mathrm{ref}}>1$ organizes the local geometry outside and provides the reference guidance layer for boundary following.

On $\gamma_{l}\left(x\right)=1$, one has $\beta_{b}\left(x\right)=0$ and $\nabla_{x}\beta_{b}\left(x\right)=0$; therefore,

$$\Gamma_{t}\left(x,t\right)=\gamma_{l}\left(x\right),\;\nabla_{x}\Gamma_{t}\left(x,t\right)=\nabla_{x}\gamma_{l}\left(x\right).$$

Thus, the two layers are consistent in both value and normal direction on the inner safety manifold.

**Proposition** **1** (Spatial $C^{2}$ smoothness of the dual-layer manifold field)**.** *At time t, suppose that the weight functions $\alpha_{i}\left(x\right)$ are $C^{2}$ with respect to the spatial variable x and satisfy*

$$\sum_{i=1}^{N_{s}}\alpha_{i}\left(x\right)>0,\;\forall x\in R^{2}.$$

*If the local quadratic form*

$${\left(x-c_{i}\right)}^{T}A_{i}\left(t\right)\left(x-c_{i}\right)$$

*is $C^{2}$ with respect to x, and if $\gamma_{l}\left(x\right)>0$ holds in the domain of definition, then the functions constructed by Equations* (48)–(51)*, namely*

$${\tilde{\Gamma }}_{t}\left(x,t\right),\;\gamma_{l}\left(x\right),\;\beta_{b}\left(x\right),\;\Gamma_{t}\left(x,t\right),$$

*are all $C^{2}$ with respect to x.*

**Proof.**  At time *t*, $\alpha_{i}\left(x\right)$ and ${\left(x-c_{i}\right)}^{T}A_{i}\left(t\right)\left(x-c_{i}\right)$ belong to $C^{2}$ with respect to *x*. Therefore, both ${\tilde{\Gamma }}_{t}\left(x,t\right)$ and $\gamma_{l}\left(x\right)$ can be written as quotients of sums of $C^{2}$ functions, where the denominator is strictly positive and twice continuously differentiable. Hence, both functions belong to $C^{2}$ with respect to *x*.

Furthermore, since $\gamma_{l}\left(x\right)>0$, one has $1/\gamma_{l}\left(x\right)\in C^{2}$, and thus

$$\eta_{b}\left(x\right)=1-\frac{1}{\gamma_{l}\left(x\right)}\in C^{2}.$$

Because squaring and the exponential mapping preserve $C^{2}$ regularity, $\beta_{b}\left(x\right)\in C^{2}$.

Finally, by Equation (51), $\Gamma_{t}\left(x,t\right)$ is a linear combination and product of $C^{2}$ functions. Therefore, $\Gamma_{t}\left(x,t\right)\in C^{2}$.    □

**Assumption** **1** (Local regularity of the outer guidance manifold)**.** *At time t, the outer guidance manifold used for modulation is represented by the scalar field $\Gamma_{t}\left(x,t\right)$. In the local neighborhood of this manifold,*

$$∥\nabla_{x}\Gamma_{t}\left(x,t\right)∥>0.$$

The satisfaction of this assumption depends on the boundary sampling density, the scale of the weighting functions, and the choice of the anisotropic parameters. If the samples are too sparse or the fused level sets become strongly corrugated, the neighborhood of the outer guidance manifold may contain very small gradients or local critical points. Therefore, for practical use, we recommend offline validation of the selected sampling and parameter settings by checking the minimum gradient norm over the modulation region. A near-zero value indicates a violation of this assumption and is outside the claimed operating scope of the proposed modulation method.

For online implementation, the proposed method evaluates the manifold fields only at the query points required by the current control update. Let $N_{s}$ denote the number of sampled NFZ boundary points. Since each field evaluation fuses these samples through weighted summation, the dominant online computational cost scales linearly with $N_{s}$, i.e., $O(N_{s})$.

**Figure 4:**
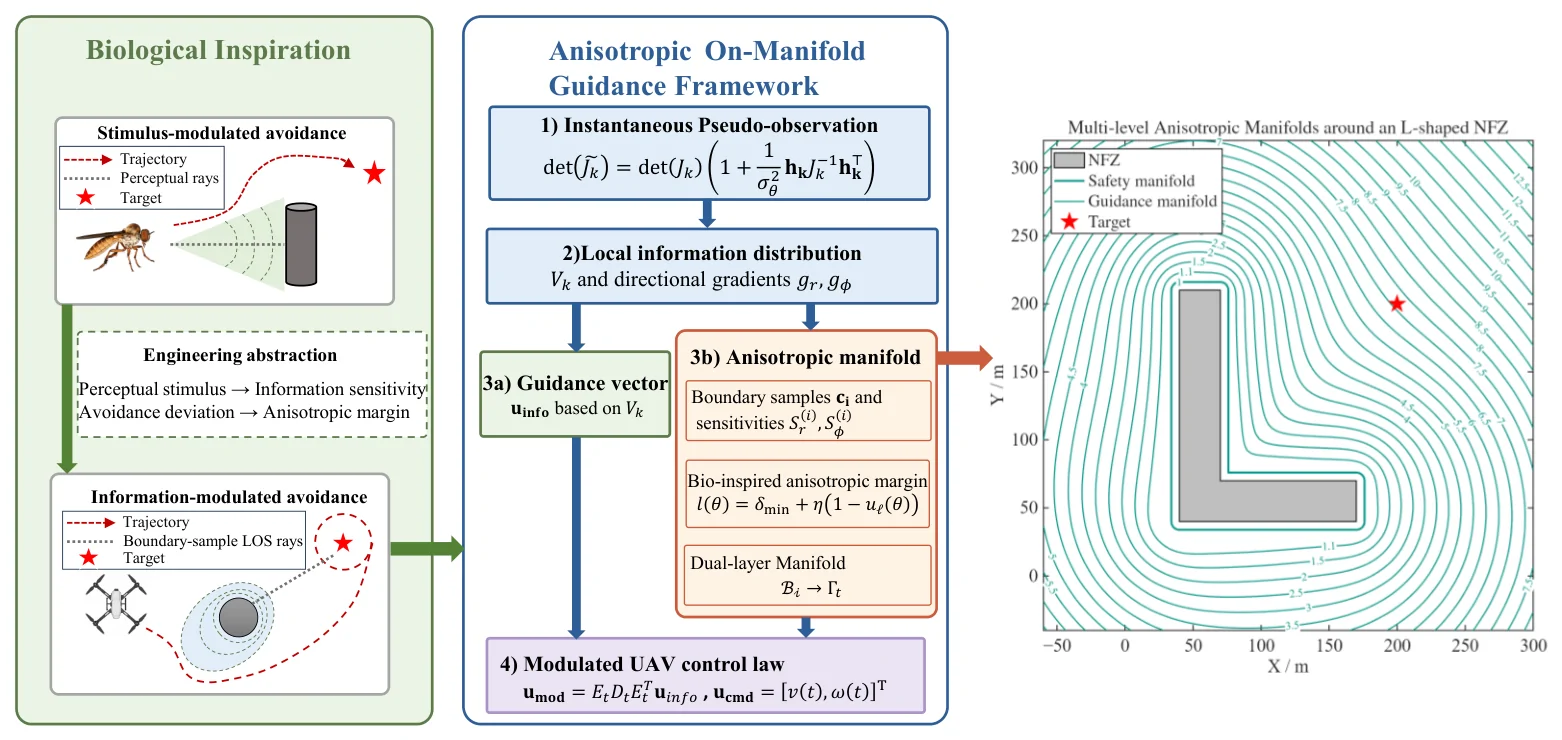
Bio-inspired interpretation and framework of the proposed anisotropic manifold guidance method.

As illustrated in Figure 4, the proposed framework establishes a bio-inspired correspondence between directional perceptual stimulus and anisotropic manifold construction. Specifically, the local information sensitivity induced by bearing-only target localization is interpreted as a task-relevant perceptual stimulus, which determines the direction-dependent margin used to construct the anisotropic manifold near the obstacle. The middle panel summarizes the corresponding algorithmic flow, while the right panel shows the resulting level sets around an L-shaped nonconvex NFZ, where the contour labels indicate the distance scale $\sqrt{\Gamma_{t}}$.

### 4.2. On-Manifold Modulation

After obtaining the time-varying anisotropic manifold outside the boundary, the next step is to construct a modulation matrix that is consistent with this geometry. The system enters NFZ avoidance mode in the neighborhood $\Gamma_{t}\left(x,t\right)\leq \gamma_{\mathrm{on}}$. All subsequent modulation uses the outer guidance manifold $\Gamma_{t}\left(x,t\right)>1$ to define the local normal–tangential basis, while the safety constraint is specified only by the inner safety manifold $\gamma_{l}\left(x\right)$. This construction is used together with the speed gate to establish forward invariance of the inner safety set defined by the inner safety manifold $\gamma_{l}\left(x\right)=1$.

Let

(52) $$n_{t}\left(x\right)=\frac{\nabla_{x}\Gamma_{t}\left(x,t\right)}{∥\nabla_{x}\Gamma_{t}\left(x,t\right)∥},\;t_{t}\left(x\right)=R_{\pi /2}n_{t}\left(x\right)$$

where $R_{\pi /2}$ denotes the counterclockwise rotation operator by π/2 in the two-dimensional plane. In the local orthonormal basis $E_{t}\left(x\right)=\left[\;n_{t}\left(x\right),\;t_{t}\left(x\right)\;\right],$ this paper adopts the following lower triangular modulation matrix [30]:

(53) $$D_{t}\left(x,t\right)=\left[\begin{array}{cc} \lambda_{n}\left(x,t\right) & 0\\ d_{tn}\left(x,t\right) & \lambda_{t}\left(x,t\right) \end{array}\right].$$

The modulated guidance vector is written as

(54) $${\bar{u}}_{\mathrm{mod}}\left(t\right)=E_{t}\left(x\right)\;D_{t}\left(x,t\right)\;E_{t}^{T}\left(x\right)\;u_{\mathrm{info}}\left(t\right).$$

A fixed reference guidance layer parameter $\gamma_{\mathrm{ref}}>1$ is used to define the reference guidance layer $\Gamma_{t}\left(x,t\right)=\gamma_{\mathrm{ref}}.$

The normal guidance term is designed as

(55) $$\lambda_{n}\left(x,t\right)=\left(1-\frac{\gamma_{\mathrm{ref}}}{\Gamma_{t}\left(x,t\right)}\right)s_{n}\left(x,t\right)$$

where $s_{n}\left(x,t\right)$ is a piecewise sign correction term acting only in the normal channel and is defined by

(56) $$s_{n}\left(x,t\right)=\left\{\begin{array}{cc} -1, & \Gamma_{t}\left(x,t\right)<\gamma_{\mathrm{ref}}\;\mathrm{and}\;n_{t}^{T}\left(x\right)u_{\mathrm{info}}\left(t\right)>0,\\ 1, & \mathrm{otherwise}. \end{array}\right.$$

Since $1-\gamma_{\mathrm{ref}}/\Gamma_{t}\left(x,t\right)$ vanishes on $\Gamma_{t}\left(x,t\right)=\gamma_{\mathrm{ref}}$, the modulated normal velocity remains zero on the reference guidance layer. Moreover, although $s_{n}\left(x,t\right)$ is piecewise, the product $s_{n}\left(x,t\right)n_{t}^{T}\left(x\right)u_{\mathrm{info}}\left(t\right)$ remains continuous and equal to zero when $n_{t}^{T}\left(x\right)u_{\mathrm{info}}\left(t\right)=0$.

For $\Gamma_{t}\left(x,t\right)<\gamma_{\mathrm{ref}}$, the sign correction redirects a nonzero nominal normal component outward toward the reference guidance layer, whereas outside the reference guidance layer the nominal normal tendency is retained. Meanwhile, on the inner safety manifold $\gamma_{l}\left(x\right)=1$, the above construction constrains the normal velocity to be nonnegative, thereby preventing the trajectory from penetrating into the unsafe region $\gamma_{l}\left(x\right)<1$.

The tangential scaling term is defined as

(57) $$\lambda_{t}\left(x,t\right)=1+\frac{1}{\Gamma_{t}\left(x,t\right)}$$

which retains the standard tangential scaling for boundary passability.

Furthermore, to provide additional tangential guidance when the nominal control vector points toward the obstacle interior, the coupling term is defined as

(58) $$d_{tn}\left(x,t\right)=-\frac{\delta_{\mathrm{tn}}}{\Gamma_{t}\left(x,t\right)}\;\Psi \;\left(-\langle u_{\mathrm{info}}\left(t\right),\;n_{t}\left(x\right)\rangle \right)$$

where $\delta_{\mathrm{tn}}>0$ is the coupling gain.

This paper sets

(59) $$\Psi \left(z\right)={\left(\max(0,z)\right)}^{p},\;p\geq 1.$$

Thus,

(60) $$d_{tn}\left(x,t\right)=-\frac{\delta_{\mathrm{tn}}}{\Gamma_{t}\left(x,t\right)}{\left(\max(0,-\langle u_{\mathrm{info}}\left(t\right),n_{t}\left(x\right)\rangle )\right)}^{p}.$$

The diagonal terms define the normal reference guidance layer correction and the tangential boundary scaling, while the off-diagonal term $d_{tn}\left(x,t\right)$ provides the information-conditioned tangential injection.

Since $\lambda_{n}\to 1$, $\lambda_{t}\to 1$, and $d_{tn}\to 0$ as $\Gamma_{t}\left(x,t\right)\to \infty$, Equation (54) asymptotically reduces to $u_{\mathrm{info}}\left(t\right)$ away from the NFZs. The modulated vector defines the desired heading as

(61) $$\psi_{\mathrm{cmd}}\left(t\right)=\arctan\left({\bar{u}}_{\mathrm{mod},y}\left(t\right),{\bar{u}}_{\mathrm{mod},x}\left(t\right)\right),$$

and the heading error is

(62) $$e_{\psi }\left(t\right)=\arctan\left(\sin\left(\psi_{\mathrm{cmd}}\left(t\right)-\psi \left(t\right)\right),\cos\left(\psi_{\mathrm{cmd}}\left(t\right)-\psi \left(t\right)\right)\right).$$

Let

(63) $$h_{l}\left(x\right)=\gamma_{l}\left(x\right)-1,\;a_{l}\left(x,\psi \right)=\nabla_{x}\gamma_{l}{\left(x\right)}^{T}g\left(\psi \right)$$

where $h_{l}\left(x\right)$ measures the signed safety level, and $a_{l}\left(x,\psi \right)$ is the normal velocity coefficient of the UAV heading direction. When $a_{l}\left(x,\psi \right)<0$, the current heading points toward the unsafe side.

The nominal speed is

(64) $$v_{\mathrm{nom}}\left(t\right)=v_{\max}\max\left\{0,\cos e_{\psi }\left(t\right)\right\}.$$

The safety speed bound is defined as

(65) $$v_{l}\left(x,\psi \right)=\left\{\begin{array}{cc} -\frac{k_{l}h_{l}\left(x\right)}{a_{l}\left(x,\psi \right)}, & a_{l}\left(x,\psi \right)<0,\\ v_{\max}, & a_{l}\left(x,\psi \right)\geq 0, \end{array}\right.\;k_{l}>0,$$

where $k_{l}$ is a safety speed gain.

The UAV input is then given by

(66) $$\left\{\begin{array}{cc} v(t) & =\min\{v_{\mathrm{nom}}\left(t\right),v_{l}\left(p_{U}\left(t\right),\psi \left(t\right)\right)\},\\ \omega (t) & ={\mathrm{sat}}_{\left[-\omega_{\max},\;\omega_{\max}\right]}\left(k_{\psi }e_{\psi }\left(t\right)\right) \end{array}\right.$$

where $k_{\psi }>0$ is the heading gain.

Thus, the final control law for the UAV model is

(67) $$u_{\mathrm{cmd}}\left(t\right)={\left[v\left(t\right),\omega \left(t\right)\right]}^{T}.$$

The implemented UAV inputs are bounded by construction. In particular, the speed is bounded by $v_{\max}$, so the maximum displacement over a finite mission horizon *T* is no larger than $v_{\max}T$. Unlike conventional dynamical systems with a fixed attractor, where the nominal vector field is designed to converge to a fixed target point, the proposed guidance field is updated online from the current state estimate and $V_{k}$ to generate informative UAV-target relative motion for reducing localization error. Therefore, this work does not claim global asymptotic stability to a fixed UAV equilibrium.

**Proposition** **2** (Forward invariance of the inner safety set)**.** *Let*

$$C_{l}=\left\{x\in R^{2}:\gamma_{l}\left(x\right)\geq 1\right\}$$

*denote the inner safety set. Under the regularity requirements for the manifold representation function given by Equations* (7) *and* (8)*, consider the UAV kinematic model in Equation* (1)* with the input defined by Equations* (61)–(66)*. If the initial condition satisfies*

$$p_{U}\left(0\right)\in C_{l},$$

*then the resulting trajectory satisfies*

$$p_{U}\left(t\right)\in C_{l},\;\forall t\geq 0.$$

*That is, the inner safety set $C_{l}$ is forward invariant, and the modulated trajectory does not penetrate from outside the inner safety manifold $\gamma_{l}\left(x\right)=1$ into the region $\gamma_{l}\left(x\right)<1$.*

The proof is given in Appendix A.

**Remark** **3** (Scope of the safety guarantee)**.** *The forward invariance result in Proposition 2 is established for the continuous-time UAV kinematic model. A sampled-data implementation introduces zero-order hold and command tracking errors. As a consequence, the safety guarantee does not transfer automatically to a discretized execution layer. In the implementation, the modulation is activated in a neighborhood outside the inner safety manifold. For the anisotropic field, the local normal distance from a dominant boundary sample to the level $\Gamma_{t}=\gamma$ is conservatively approximated by $d\left(\gamma \right)\approx \delta_{\min}\sqrt{\gamma }$, since its direction-dependent effective scale is no smaller than $\delta_{\min}$. Considering the worst-case one-step inward motion, whose normal displacement is bounded by $v_{\max}\Delta T$, and neglecting the additional speed reduction induced by the safety speed gate, a practical buffer guideline is*

$$\delta_{\min}\left(\sqrt{\gamma_{\mathrm{on}}}-1\right)\geq v_{\max}\Delta T,\;\gamma_{\mathrm{on}}\geq {\left(1+\frac{v_{\max}\Delta T}{\delta_{\min}}\right)}^{2}.$$

*This condition is used as a practical parameter selection guideline for the active modulation neighborhood.*

### 4.3. Local Detour Branch Selection

The desired UAV motion is generated online by the local field $V_{k}$, which varies over time. Because its direction depends on the spatial distribution of bearing-only localization uncertainty, the tangential component of the gradient may flip during obstacle avoidance, which can cause repeated switching of the modulation direction and induce trajectory oscillations. To mitigate this effect, a local detour branch is selected and locked during each avoidance phase.

Let $\gamma_{l}\left(x\right)=1$ be the baseline safety contour, and define the set of closed baseline contours at $t_{k}$ as

(68) $$S\left(t_{k}\right)=\left\{C_{j}\left(t_{k}\right):C_{j}\left(t_{k}\right)\subset \left\{x:\gamma_{l}\left(x\right)=1\right\},\;C_{j}\left(t_{k}\right)\;\mathrm{is}\;\mathrm{closed}\right\}.$$

For a point q on an active contour, define ${\hat{l}}_{k}\left(q\right)=\left({\hat{p}}_{T}\left(t_{k}\right)-q\right)/∥{\hat{p}}_{T}\left(t_{k}\right)-q∥$ and

$$g_{k}\left(q\right)=\langle \frac{\nabla \gamma_{l}\left(q\right)}{∥\nabla \gamma_{l}\left(q\right)∥},{\hat{l}}_{k}\left(q\right)\rangle .$$

The exit point on each candidate branch is selected as the first point satisfying $g_{k}\left(q\right)>\varepsilon_{g}$; if no such point exists, the point with the largest $g_{k}\left(q\right)$ is used. The corresponding branch cost is

(69) $$J_{\sigma ,k}=L_{\sigma }+∥p_{\mathrm{ex}}^{\sigma }-{\hat{p}}_{T}\left(t_{k}\right)∥,\;\sigma \in \left\{+1,-1\right\},$$

where $L_{\sigma }$ is the contour arc length from the entry point to $p_{\mathrm{ex}}^{\sigma }$.

The complete branch selection and locking procedure at sampling instant $t_{k}$ is summarized in Algorithm 1. A hysteresis rule is used: the branch lock is activated when $\Gamma_{t}\left(p_{U}\left(t_{k}\right),t_{k}\right)\leq \gamma_{\mathrm{on}}$, and is released only after $\Gamma_{t}\left(p_{U}\left(t_{k}\right),t_{k}\right)>\gamma_{\mathrm{off}}$, with $\gamma_{\mathrm{on}}<\gamma_{\mathrm{off}}$. This avoids repeated activation and deactivation near the NFZ-interaction boundary. In the algorithm, $n_{t}=\nabla \Gamma_{t}/∥\nabla \Gamma_{t}∥$, $t_{t}=R_{\pi /2}n_{t}$, and ${\mathrm{sgn}}_{+}\left(a\right)=1$ for a≥0 and −1 otherwise.

Using the returned branch direction, the final modulated guidance vector at $t_{k}$ is written as

(70) $${\bar{u}}_{\mathrm{mod}}\left(t_{k}\right)=E_{\sigma }\left(x,t_{k}\right)D_{t}\left(x,t_{k}\right)\left[\begin{array}{cc} 1 & 0\\ 0 & \chi_{\sigma }\left(t_{k}\right) \end{array}\right]E_{\sigma }^{T}\left(x,t_{k}\right)u_{\mathrm{info}}\left(t_{k}\right),$$

where $E_{\sigma }\left(x,t_{k}\right)$ and $\chi_{\sigma }\left(t_{k}\right)$ are given by Algorithm 1. This vector is used to define the heading reference of the UAV kinematic model, and the actual input is given by Equations (61)–(66).

Since Algorithm 1 is updated only at the sampling instants $t_{k}=k\Delta T$, over any finite interval [0,T], $N_{\mathrm{sw}}\left(T\right)\leq \left\lfloor T/\Delta T\right\rfloor +1$, which rules out infinitely fast branch switching in the implemented sampled-data controller. The branch lock only fixes the tangential direction; it does not modify the normal direction, the normal eigenvalue, or the safety speed gate. Therefore, the forward invariance argument for the inner safety set is preserved.
**Algorithm 1** Local detour branch selection and locking at $t_{k}$**Input: **$p_{U}\left(t_{k}\right)$, $u_{\mathrm{info}}\left(t_{k}\right)$, ${\hat{p}}_{T}\left(t_{k}\right)$, $\gamma_{l}\left(x\right)$, $\Gamma_{t}\left(x,t_{k}\right)$, $S(t_{k})$, $\rho_{k-1}$, $\sigma_{k-1}$, $\gamma_{\mathrm{on}}$, $\gamma_{\mathrm{off}}$, $\varepsilon_{g}$, and $\varepsilon_{J}$.**Output:** branch lock flag $\rho_{k}$, branch direction $\sigma_{k}$, basis $E_{\sigma }\left(x,t_{k}\right)$, and tangential gate $\chi_{\sigma }\left(t_{k}\right)$. 1:**if **$\rho_{k-1}=0$** then** 2:    **if** $\Gamma_{t}\left(p_{U}\left(t_{k}\right),t_{k}\right)\leq \gamma_{\mathrm{on}}$ **then** 3:        Select $C_{a}\left(t_{k}\right)\leftarrow \arg\min_{C_{j}\left(t_{k}\right)\in S\left(t_{k}\right)}d\left(p_{U}\left(t_{k}\right),C_{j}\left(t_{k}\right)\right)$, and project $p_{U}\left(t_{k}\right)$ onto $C_{a}\left(t_{k}\right)$ to obtain $p_{\mathrm{en}}$. 4:         **for** σ∈{+1,−1} **do** 5:           Search from $p_{\mathrm{en}}$ along $C_{a}\left(t_{k}\right)$ in direction σ, and set $p_{\mathrm{ex}}^{\sigma }$ to the first point satisfying $g_{k}\left(q\right)>\varepsilon_{g}$; if none exists, use the searched point with the largest $g_{k}\left(q\right)$. 6:             Compute $J_{\sigma ,k}\leftarrow L_{\sigma }+∥p_{\mathrm{ex}}^{\sigma }-{\hat{p}}_{T}\left(t_{k}\right)∥$. 7:        Set $\sigma_{k}\leftarrow \arg\min_{\sigma \in \{+1,-1\}}J_{\sigma ,k}$; if $|J_{+1,k}-J_{-1,k}|\leq \varepsilon_{J}$, set $\sigma_{k}\leftarrow \arg\max_{\sigma \in \{+1,-1\}}\langle t_{\sigma }\left(p_{\mathrm{en}}\right),u_{\mathrm{info}}\left(t_{k}\right)\rangle$. 8:        Activate the branch lock, set $\rho_{k}\leftarrow 1$, and store $C_{a}\left(t_{k}\right)$ and $\sigma_{k}$. 9:    **else**10:        Set $\rho_{k}\leftarrow 0$ and $\sigma_{k}\leftarrow {\mathrm{sgn}}_{+}\;\left(u_{\mathrm{info}}^{T}\left(t_{k}\right)t_{t}\left(p_{U}\left(t_{k}\right),t_{k}\right)\right)$.11:**else**12:    Keep $\rho_{k}\leftarrow 1$ and $\sigma_{k}\leftarrow \sigma_{k-1}$.13:    **if** $\Gamma_{t}\left(p_{U}\left(t_{k}\right),t_{k}\right)>\gamma_{\mathrm{off}}$ **then**14:        Reset the branch lock, set $\rho_{k}\leftarrow 0$, and set $\sigma_{k}\leftarrow {\mathrm{sgn}}_{+}\;\left(u_{\mathrm{info}}^{T}\left(t_{k}\right)t_{t}\left(p_{U}\left(t_{k}\right),t_{k}\right)\right)$.15:Set $t_{\sigma }\left(x,t_{k}\right)\leftarrow \sigma_{k}t_{t}\left(x,t_{k}\right)$ and $E_{\sigma }\left(x,t_{k}\right)\leftarrow \left[\;n_{t}\left(x,t_{k}\right),t_{\sigma }\left(x,t_{k}\right)\;\right]$.16:Set $\chi_{\sigma }\left(t_{k}\right)\leftarrow 1$ if $\rho_{k}=0$ or $t_{\sigma }^{T}u_{\mathrm{info}}\left(t_{k}\right)\geq 0$; otherwise set $\chi_{\sigma }\left(t_{k}\right)\leftarrow 0$.17:Return $\rho_{k}$, $\sigma_{k}$, $E_{\sigma }\left(x,t_{k}\right)$, and $\chi_{\sigma }\left(t_{k}\right)$.

## 5. Simulation Results

This section evaluates the proposed method in three representative scenarios: an NFZ-free baseline case, denoted as Scenario A; a case with a nonconvex NFZ, denoted as Scenario B; and a case with dense NFZs, denoted as Scenario C. These scenarios are used to examine the ability of the method to generate informative motion, to pass around a nonconvex NFZ, and to remain effective in complex constrained environments. All simulations were implemented in MATLAB R2024a on a computer equipped with an Intel Core i7-11800H CPU.

### 5.1. Simulation Setup

The simulations consider a stationary target in a two-dimensional plane. At each sampling instant $t_{k}$, the bearing-only measurement, the EKF update, and the FIM update are performed. Then, the informative guidance field is constructed from the updated target estimate ${\hat{p}}_{T}\left(k\right)$ and the information matrix $J_{k}$, while the EKF covariance $P_{k}$ is used in the construction of the on-manifold modulation parameters. The resulting modulated guidance vector is used to define the heading reference, and the actual UAV command is $u_{\mathrm{cmd}}={\left[v,\omega \right]}^{T}$.

The isotropic on-manifold baseline follows the sample-based quadratic representation method in [30]. In this baseline, each boundary sample is assigned the same margin in all local directions, so that the local shifted boundary is a circle with radius $l_{\mathrm{iso}}$. The corresponding isotropic manifold field is defined as

(71) $$\Gamma_{\mathrm{iso}}\left(x\right)=\frac{\sum_{i=1}^{N_{s}}\alpha_{i}\left(x\right){∥x-c_{i}∥}^{2}/l_{\mathrm{iso}}^{2}}{\sum_{i=1}^{N_{s}}\alpha_{i}\left(x\right)}.$$

This baseline shares the same guidance law, on-manifold modulation, branch-selection mechanism, and other implementation parameters as the proposed method, except that the anisotropic manifold field is replaced by $\Gamma_{\mathrm{iso}}\left(x\right)$.

A basic CBF-QP baseline [42] is also included:

(72) $$\begin{array}{cc} u_{\mathrm{CBF}} & =\arg\min_{∥u∥\leq v_{\max}}{∥u-v_{\max}u_{\mathrm{info}}∥}^{2},\\ s.t. & \nabla h_{j}{\left(p_{U}\right)}^{T}u+\alpha_{\mathrm{CBF}}h_{j}\left(p_{U}\right)\geq 0,\;j=1,\ldots ,N_{o},\\ \; & h_{j}\left(p_{U}\right)=d_{j}\left(p_{U}\right)-d_{\mathrm{safe}}^{\mathrm{CBF}}. \end{array}$$

where $d_{j}\left(p_{U}\right)$ is the signed distance to $\partial O_{j}$, taken positive outside the *j*-th NFZ. In this baseline, the constraints are imposed for all NFZs and the signed distances are computed from the same sampled NFZ boundaries used by the proposed method.

The common parameters used in all simulations are summarized in Table 1. Additional scenario settings, including target positions and UAV initial states, are described in the corresponding subsections. In all simulation cases, no zero-gradient point was observed over the modulation regions.

### 5.2. Results and Analysis

#### 5.2.1. Scenario A: Baseline Target Localization in an NFZ-Free Environment

This experiment compares the proposed perception-guided control law with two baseline methods in an NFZ-free environment, where the baselines do not include an NFZ avoidance mechanism. For the Deghat baseline [25], we used the bearing-only circumnavigation controller with d=50m and $b_{D}=7$. For the Yang baseline [19], we used a single-step receding-horizon implementation, i.e., $T_{p}=1$. All baselines shared the same EKF, noise model, sampling period, initial conditions, and UAV kinematic limits. In this scenario, the true target position is set to (400,400)m, while the UAV is initialized at (20,20)m with an initial heading angle of $25^{\circ }$, and all other parameters are the same as those in Section 5.1.

**Figure 5:**
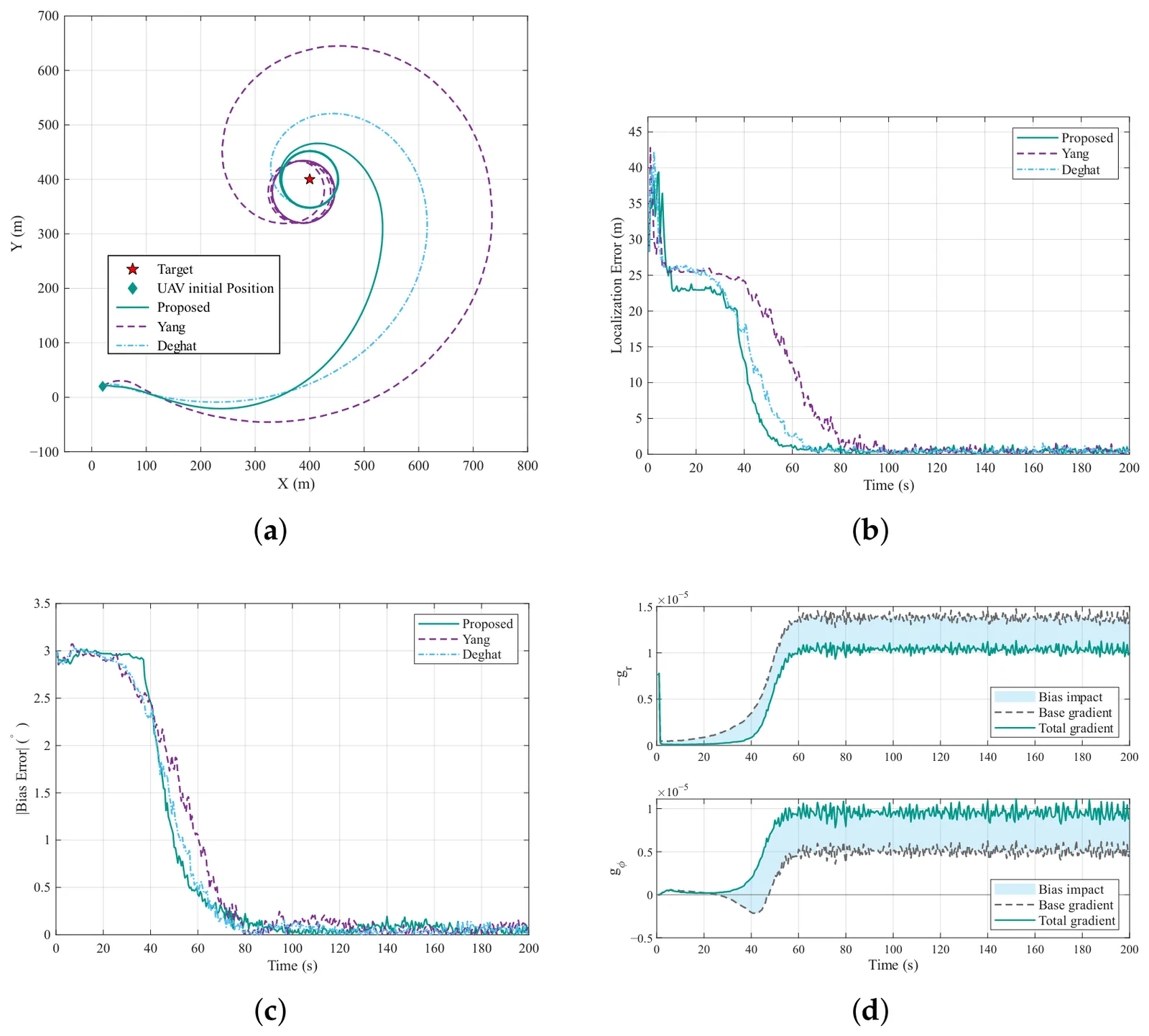
Scenario A: Comparison of trajectories and estimation errors for the three methods in the NFZ-free environment. (**a**) UAV trajectories. (**b**) Position estimation error. (**c**) Bias estimation error. (**d**) Evolution of $g_{r}$ and $g_{\phi }$.

Figure 5a shows that the proposed method produces a more compact spiral trajectory. At the beginning, the UAV is far from the target. As a result, the radial contraction is relatively weak, while the tangential gradient $g_{\phi }$ plays a larger role in adjusting the LOS angle. The trajectory therefore shows a clear tangential motion at first, so that the observation direction gradually moves toward a more favorable geometric configuration. As the sensor bias estimate converges, the radial contraction becomes stronger, and the trajectory enters a spiral approach phase with a steadily decreasing radius.

Compared with the method of Deghat et al., the proposed method adjusts the tangential bearing geometry earlier, which leads to faster convergence in the intermediate stage. The method of Yang et al. combines the radial, tangential, and bias effects into a single scalar objective. When the bias has not yet converged sufficiently, it tends to maintain tangential motion for a longer time, which results in a larger orbit radius and a longer path before convergence. By contrast, the proposed method starts to contract inward earlier after the initial adjustment of the LOS angle, which leads to a more compact trajectory. As shown in Figure 5b,c, all three methods exhibit a slow initial error reduction due to the combined effect of the initial observation geometry and sensor bias, while the proposed method achieves earlier convergence of the position error.

Figure 5d further supports the theoretical analysis from the perspective of the gradient. The radial gradient $g_{r}$ remains negative throughout the process, which indicates that the system always keeps a basic tendency to contract toward the neighborhood of the target. However, during the initial long-range stage, the bias coupling term, shown by the shaded region, partly weakens the radial contraction. This prevents the trajectory from entering a purely monotonic radial approach too early. At the same time, the tangential gradient $g_{\phi }$ is more dominant at the beginning, which drives the LOS angle toward a more favorable observation geometry. The bias coupling term further increases the tangential gradient, which requires a larger tangential maneuver and produces a more pronounced spiral convergence trend.

Scenario A was further evaluated using 50 independent Monte Carlo runs with resampled bearing noise. Both the biased setting and a bias-free setting were tested. In the bias-free setting, the EKF state contains only the target position. Here $e_{p}\left(t_{k}\right)=∥{\hat{p}}_{T}\left(t_{k}\right)-p_{T}\left(t_{k}\right)∥$ denotes the position error, and $e_{b}\left(t_{k}\right)=\hat{b}\left(t_{k}\right)-b$ denotes the signed bearing-bias error. In the following Monte Carlo tables, each entry reports the mean and standard deviation over independent runs. The Mean $|e_{b}|$ denotes the time-averaged absolute bias error, while Final $e_{b}$ denotes the signed final bias error.

As shown in Table 2, the proposed method yields the lowest mean position RMSE in the presence of bearing bias and remains comparable to the reference methods without bearing bias.

#### 5.2.2. Scenario B: Target Localization Under Nonconvex NFZ Constraints

This experiment evaluates the NFZ avoidance capability of the proposed anisotropic on-manifold guidance strategy under nonconvex NFZ constraints. In this scenario, the true target position is set to (600,600)m, while the UAV is initialized at (0,0)m with an initial heading angle of $25^{\circ }$, and all other parameters are the same as those in Section 5.1.

**Figure 6:**
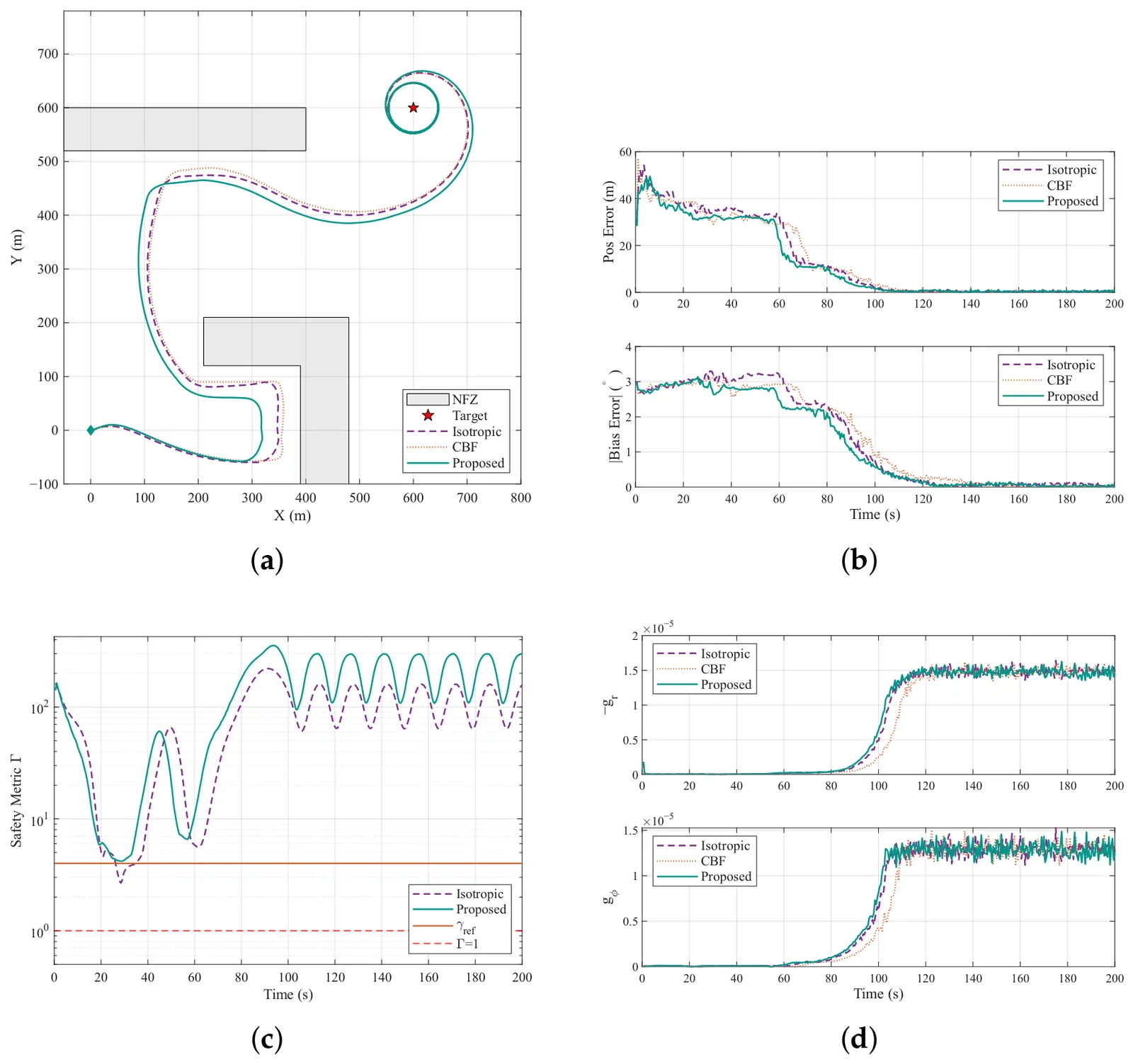
Scenario B: Trajectories, estimation performance, and safety-related quantities in the nonconvex NFZ environment. (**a**) Trajectory comparison in the nonconvex NFZ field. (**b**) Estimation errors under NFZ-constrained motion. (**c**) Safety metric during NFZ avoidance. (**d**) Evolution of $g_{r}$ and $g_{\phi }$.

As shown in Figure 6, when the UAV approaches the NFZ boundary, the proposed method produces a more evident outward expansion along directions with lower information sensitivity, which corresponds to a larger margin during avoidance. When the UAV enters a region with higher information sensitivity, the anisotropy of the manifold gradually weakens. Consequently, the added margin is reduced, and the trajectory takes a more compact detour around the NFZ, approaching the isotropic baseline behavior. The CBF baseline directly projects the $u_{\mathrm{info}}$ into local geometric safety constraints, which produces a slower reduction in the position and bias errors compared with the proposed method. Figure 6c shows the evolution of the manifold metric. In the simulated NFZ avoidance stage, the proposed method regulates the motion in the vicinity of the guidance layer. As the trajectory approaches this layer, the normal component of the velocity gradually decreases and converges, indicating that the modulated vector field tends to align the motion with the local tangential direction.

Figure 6d shows that, because the outward detour is more conservative at the beginning, the UAV enters a region with more favorable information sensitivity earlier. As a result, the local information gradient increases slightly, which leads to an earlier reduction in the localization error.

**Figure 7:**
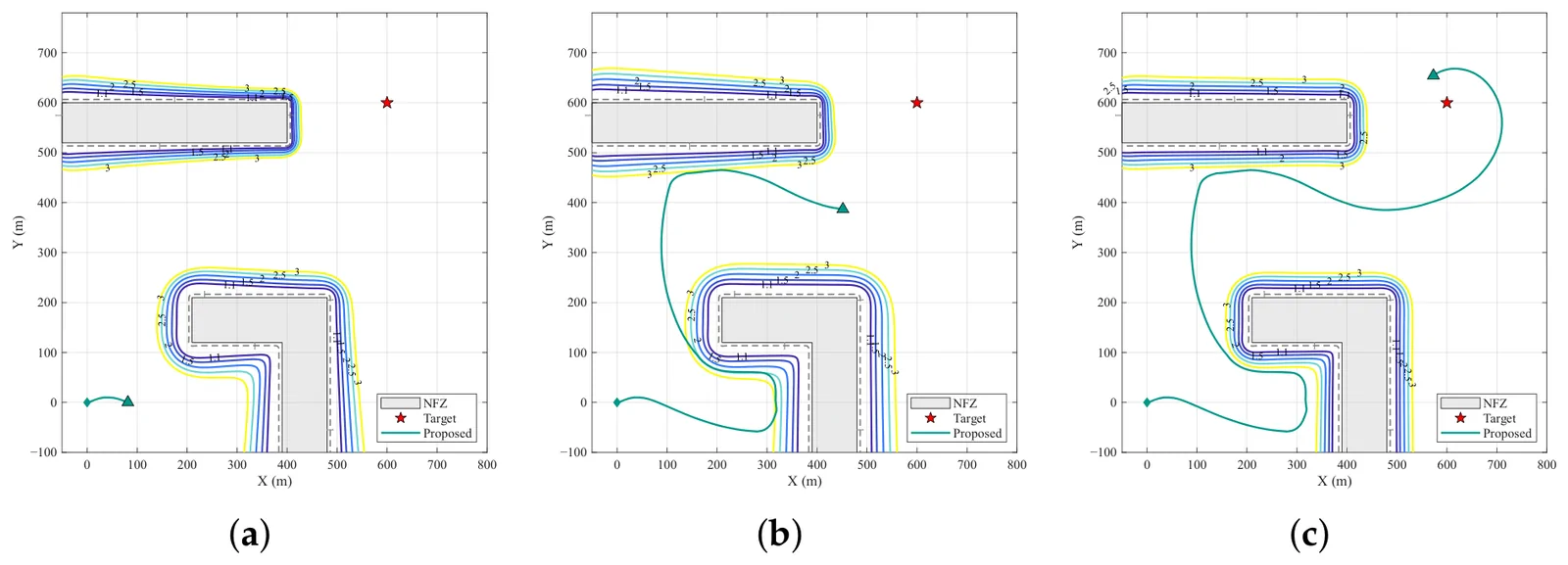
Scenario B: Temporal evolution of the anisotropic manifold field in the nonconvex NFZ scenario. (**a**) t=5s. (**b**) t=70s. (**c**) t=100s.

Figure 7 illustrates the dual-layer manifolds constructed around the NFZs. The gray dashed contours denote the inner safety manifolds, while the colored contours show the adaptive outer guidance manifolds at three representative instants. The manifold was well defined in this scenario, with the minimum of $∥\nabla_{x}\Gamma_{t}∥$ equal to 0.197. At the initial stage, t=5s, the estimate of the target state is still inaccurate, and the anisotropic expansion is stronger in regions with lower information sensitivity. The manifold boundary therefore shows a clearly nonuniform outward expansion, with a thicker region on the far side and a thinner region on the near side. As the UAV continues to collect measurements and gradually establishes a stable observation geometry, the anisotropy of the corresponding manifold weakens. At t=70s, the directional difference in the boundary has largely converged. At t=100s, the estimation error approaches its steady state, and the manifold gradually degenerates into an approximately isotropic shape.

**Figure 8:**
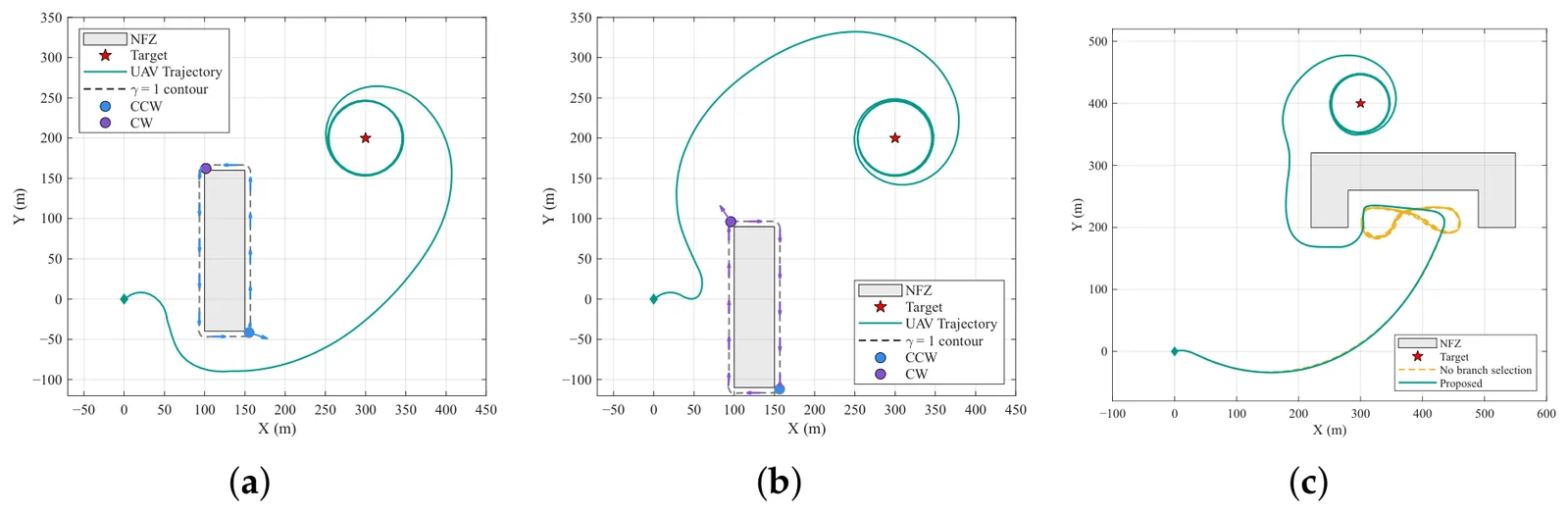
Scenario B: Representative branch selections, with the arrow indicating the selected branch direction. (**a**) Counterclockwise branch. (**b**) Clockwise branch. (**c**) Trajectories with and without branch selection.

Figure 8a,b compare the candidate detour branches under two different relative configurations between the UAV and the nonconvex NFZ in Scenario B. In Figure 8a, the counterclockwise (CCW) branch has a shorter contour arc length to its exit point and a smaller distance from the exit point to the estimated target, yielding a lower total detour cost $J_{\sigma }$. Therefore, the CCW branch is selected. In Figure 8b, the clockwise (CW) branch has both a shorter arc length and an exit point whose outward normal is more nearly perpendicular to the LOS, indicating a more favorable observation geometry at departure. Therefore, the CW branch is selected for circumnavigation. The proposed branch selection criterion jointly minimizes the contour arc length and the distance from the exit point to the estimated target, and the selected branch is locked throughout the avoidance phase, suppressing repeated switching caused by local fluctuations in $V_{k}$ or heading perturbations. As shown in Figure 8c, the absence of branch selection induces oscillatory motion near the concave passage.

#### 5.2.3. Scenario C: Target Localization Under Dense NFZ Environments

**Figure 9:**
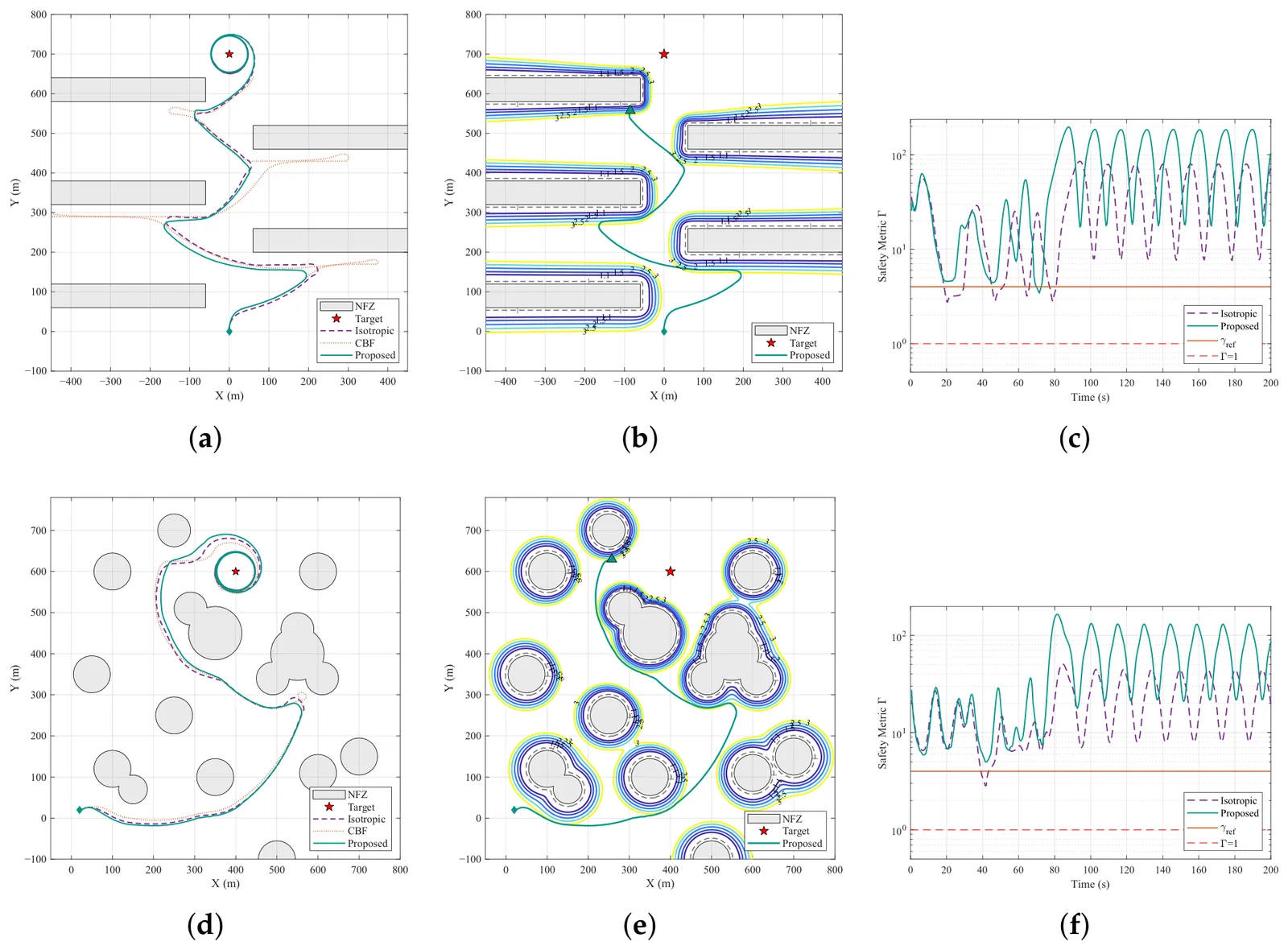
Scenario C: Dense NFZ evaluation under two NFZ configurations. (**a**) UAV trajectories in Scenario C1. (**b**) Local manifold field in Scenario C1. (**c**) Safety metric in Scenario C1. (**d**) UAV trajectories in Scenario C2. (**e**) Local manifold field in Scenario C2. (**f**) Safety metric in Scenario C2.

The first row of Figure 9 corresponds to a structured dense NFZ environment (Scenario C1), where the true target position is set to (0,700)m and the UAV is initialized at (0,0)m with an initial heading angle of $90^{\circ }$. The second row corresponds to a clustered circular dense NFZ environment (Scenario C2), where the true target position is set to (400,600)m and the UAV is initialized at (20,20)m with an initial heading angle of $0^{\circ }$. All other parameters are the same as those in Section 5.1. Each row presents the trajectory, a local slice of the manifold field, and the safety metric for the corresponding case.

The trajectory results show that the proposed method maintains effective guidance and NFZ avoidance across both scenarios. It preserves the tangential motion tendency near NFZ boundaries while avoiding penetration of the inner safety manifold. The slices of the manifold field further illustrate that the proposed information-shaped field produces locally anisotropic deformation around the NFZs. In Scenario C1, the CBF baseline shows a longer detour and a late turn-back behavior. This is mainly caused by its local minimum-norm projection of $u_{\mathrm{info}}$, which does not include the branch selection used in the proposed method.

The manifolds were well defined in these cases, with the minimum values of $∥\nabla_{x}\Gamma_{t}∥$ equal to 0.274 and 0.208 for Scenario C1 and Scenario C2, respectively. The curves of the manifold metric show that the proposed trajectory remains above the inner safety manifold during the main NFZ avoidance stage. When the trajectory approaches the reference guidance layer, the normal component of the velocity is attenuated by the proposed modulation. The actual safety constraint is evaluated by the inner safety manifold $\gamma_{l}\left(x\right)=1$, and no penetration of this inner safety manifold is observed.

The localization performance statistics for Scenario C1 and Scenario C2 are given in Table 3. Each entry reports the mean and standard deviation over 50 independent runs with resampled bearing noise. In both cases, the proposed method achieves lower estimation errors than the other methods.

Overall, the results of Scenario C show that the proposed method can maintain local guidance and NFZ avoidance in both the structured dense NFZ case and the clustered circular dense NFZ case.

Under the sampled UAV kinematic execution with ΔT=0.5s, the mean heading deviations were $11.505^{\circ }\pm 0.462^{\circ }$ in Scenario C1 and $6.586^{\circ }\pm 0.070^{\circ }$ in Scenario C2. The worst values of $\min\Gamma_{t}$ were 3.405 and 4.929, respectively, and the corresponding worst physical clearances were 20.845m and 27.437m. These results show that, under the nominal sampling period used in this work, the UAV kinematic model can execute the modulated guidance command with acceptable heading error while maintaining positive physical clearance from the NFZ boundaries.

Table 4 reports the online computation time in the dense NFZ cases, including the time cost of branch selection. The maximum online computation time remains below the sampling period ΔT=500ms in all reported cases.

To quantify the effect of the branch selection module, an ablation study was conducted in the two dense NFZ cases. The variant without branch selection uses the same anisotropic field and modulation matrix as the proposed method, but disables the branch choice and branch lock. The oscillation count is defined as $N_{\mathrm{osc}}=\sum_{k\in K}I\left(s_{k}s_{k-1}<0\right)$, where $s_{k}=\mathrm{sign}\left(t_{t}{\left(p_{U}\left(t_{k}\right),t_{k}\right)}^{⊤}{\bar{u}}_{\mathrm{mod}}\left(t_{k}\right)\right)$, $K=\{k:\Gamma_{t}\left(p_{U}\left(t_{k}\right),t_{k}\right)\leq \gamma_{\mathrm{off}}\}$, I(·) is the indicator function.

**Figure 10:**
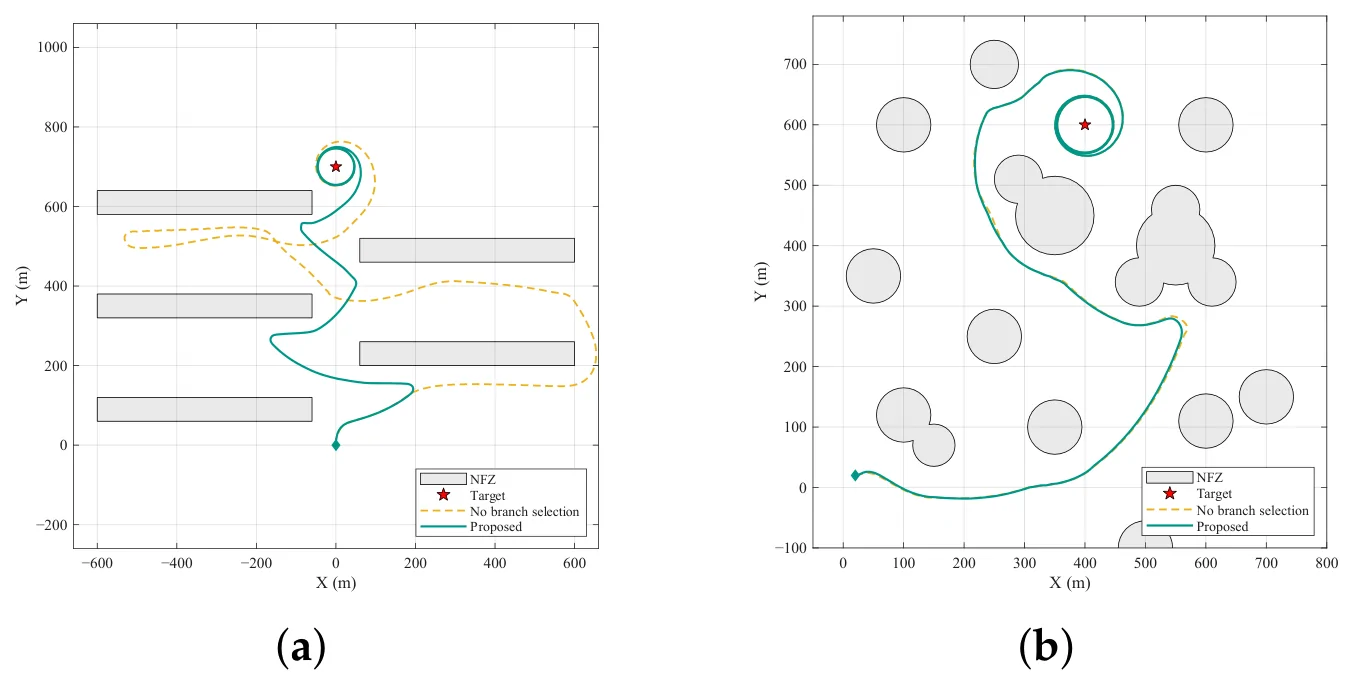
Branch selection ablation trajectories: (**a**) Scenario C1 and (**b**) Scenario C2.

As shown in Table 5, branch selection reduces $N_{\mathrm{osc}}$ from 6 to 3 and lowers the position RMSE from 19.892m to 17.041m in Scenario C1. In Scenario C2, the oscillation count is already low for both variants, but the proposed method still gives a lower position RMSE and a larger worst minimum $\Gamma_{t}$. These results show that the branch lock reduces repeated changes in the detour direction in the tighter case and improves the robustness of the detour behavior. The corresponding trajectories are shown in Figure 10.

Parameter sensitivity is evaluated in Scenarios C1 and C2 by varying each parameter separately, while keeping the remaining parameters at their nominal values. As shown in Table 6, the selected nominal values provide a balanced compromise between localization accuracy and safety clearance in the two representative configurations. Among these parameters, $\kappa_{\beta }$ shows no noticeable influence on performance, as it only governs the narrow blending region near the inner safety layer.

## 6. Conclusions

This paper studies bearing-only UAV target localization under NFZ constraints by jointly considering information acquisition and NFZ avoidance. A perception-guided control law was developed from a projected uncertainty field constructed by an instantaneous pseudo-observation, so that the UAV motion can be adjusted according to the local observation geometry. To incorporate NFZ avoidance into this perception-guided motion, the local information sensitivity was further proposed to shape an anisotropic dual-layer manifold field around the NFZs. Based on this field, an on-manifold modulation strategy was designed, thereby enabling NFZ detouring while retaining the tendency to improve the bearing-only localization geometry. Simulation results from NFZ-free, nonconvex NFZ, and dense NFZ scenarios show that the proposed method can generate NFZ detouring trajectories while achieving favorable estimation performance.

Several limitations will be considered in future work. The proposed on-manifold modulation relies on proper sampling and parameter settings to satisfy Assumption 1; these settings were checked offline in the reported simulations. Future work will consider real-time regularity monitoring and parameter adaptation for dense obstacle environments. A complete sampled-data safety analysis with explicit tracking error bounds also remains for future work. The framework will be further extended to moving targets, three-dimensional UAV guidance with volumetric NFZ constraints, and time-varying or uncertain restricted regions.

## Figures and Tables

**Table 1:** Simulation parameters.

Parameter Group	Value
Simulation horizon	ΔT=0.5s, $N_{\mathrm{sim}}=400$.
State initialization	$b_{0}=3^{\circ }$, ${\hat{b}}_{0}=0^{\circ }$; ${\hat{p}}_{T,0}=p_{T}+\left(-20,-20\right)\;m$; $P_{0}=\mathrm{diag}\left(500,500,{\left(0.5^{\circ }\right)}^{2}\right)$.
Measurement and process noise	$\sigma_{\theta }=0.5^{\circ }$; $Q_{k}=\mathrm{diag}\left(0.5\;m^{2},0.5\;m^{2},{\left(0.01^{\circ }\right)}^{2}\right)$.
UAV motion parameters	$v_{\max}=20\;m/s$, $\omega_{\max}=25^{\circ }/s$, $k_{\psi }=2.0$, $k_{r}=0.3$, $R_{\mathrm{safe}}=50\;m$, $R_{w}=40\;m$, $k_{l}=1.0\;s^{-1}$.
Manifold construction	h=5m, $\gamma_{\mathrm{on}}=12$, $\gamma_{\mathrm{off}}=13$, n=4, $\kappa =10^{5}$, $\kappa_{\beta }=500$, $\mu_{s}=10$, $\delta_{\min}=10\;m$.
Modulation parameters	$\eta_{\min}=1$, $\eta_{\max}=80$, $k_{c}=3$; $\gamma_{\mathrm{ref}}=4$, $\delta_{\mathrm{tn}}=20.0$, p=2, $\varepsilon_{g}=0.05$.
Numerical regularization parameters	$\varepsilon_{u}=\varepsilon_{w}=\varepsilon_{J}=10^{-10}$.
Isotropic baseline	$l_{\mathrm{iso}}=30\;m$.
CBF baseline	$d_{\mathrm{safe}}^{\mathrm{CBF}}=30\;m$, $\alpha_{\mathrm{CBF}}=0.45$.

**Table 2:** Monte Carlo localization performance in Scenario A over 50 runs.

*With Bearing Bias*								
**Method**	**Mean $e_{p}$**	**Std. $e_{p}$**	${\mathrm{RMSE}}_{p}$	**Final $e_{p}$**	**Mean $|e_{b}|$**	**Std. $e_{b}$**	${\mathrm{RMSE}}_{b}$	**Final $e_{b}$**
	**(m)**	**(m)**	**(m)**	**(m)**	**(°)**	**(°)**	**(°)**	**(°)**
Proposed	5.809±0.155	10.087±0.343	11.629±0.372	0.482±0.319	0.746±0.016	1.150±0.010	1.369±0.011	0.048±0.058
Yang	8.345±0.214	11.046±0.371	13.832±0.422	0.365±0.259	0.842±0.023	1.145±0.021	1.421±0.029	0.047±0.063
Deghat	6.242±0.159	10.054±0.332	11.823±0.364	0.470±0.303	0.763±0.019	1.143±0.013	1.373±0.018	0.042±0.061
***Without Bearing Bias***								
**Method**	**Mean $e_{p}$**	**Std. $e_{p}$**	${\mathrm{RMSE}}_{p}$	**Final $e_{p}$**				
	**(m)**	**(m)**	**(m)**	**(m)**				
Proposed	1.721±0.279	4.695±0.715	4.996±0.758	0.480±0.271				
Yang	1.750±0.243	4.662±0.542	4.975±0.584	0.363±0.198				
Deghat	1.898±0.300	4.756±0.678	5.117±0.731	0.460±0.244				

**Table 3:** Monte Carlo localization performance in Scenario C over 50 runs.

Case	Mean $e_{p}$ (m)	Std. $e_{p}$ (m)	${\mathrm{RMSE}}_{p}$ (m)	Mean $|e_{b}|$ (°)	Std. $e_{b}$ (°)	${\mathrm{RMSE}}_{b}$ (°)
C1, proposed	10.350±0.233	13.555±0.357	17.041±0.420	1.390±0.021	1.417±0.021	1.984±0.016
C1, isotropic	11.063±0.307	13.797±0.387	17.671±0.487	1.465±0.056	1.422±0.023	2.041±0.047
C1, CBF	19.359±0.323	11.956±0.422	22.747±0.474	1.890±0.049	0.907±0.032	2.096±0.041
C2, proposed	10.084±0.299	13.858±0.370	17.124±0.472	1.233±0.039	1.274±0.029	1.772±0.042
C2, isotropic	10.547±0.279	13.995±0.248	17.510±0.364	1.243±0.140	1.285±0.046	1.789±0.123
C2, CBF	20.210±5.666	14.002±2.743	25.062±3.878	2.065±0.423	1.073±0.352	2.367±0.280

**Table 4:** Online runtime statistics in Scenario C over 50 runs.

Case	$N_{s}$	ΔT (ms)	Mean Time (ms)	Max Time (ms)	Branch Selection (ms)
C1, Proposed	1422	500	16.27±3.27	48.49±0.48	5.64±0.26
C1, Isotropic	1422	500	15.93±4.28	26.93±5.57	1.07±0.22
C1, CBF	1422	500	0.98±0.23	7.25±1.19	–
C2, Proposed	625	500	6.63±0.05	43.20±2.45	4.14±0.06
C2, Isotropic	625	500	6.31±0.03	14.53±0.21	0.51±0.01
C2, CBF	625	500	1.13±0.01	6.48±0.06	–

**Table 5:** Monte Carlo branch selection ablation in Scenario C over 50 runs.

Case	$N_{\mathrm{osc}}$	Worst min. $\Gamma_{t}$	Worst Clearance (m)	${\mathrm{RMSE}}_{p}$ (m)	${\mathrm{RMSE}}_{b}$ (°)
C1, Proposed	3.000±0.000	3.405	20.845	17.041±0.420	1.984±0.016
C1, No branch selection	6.000±0.000	3.401	31.191	19.892±0.325	1.901±0.031
C2, Proposed	2.000±0.000	4.929	27.437	17.124±0.472	1.772±0.042
C2, No branch selection	2.000±0.000	4.368	25.592	17.639±0.613	1.816±0.048

**Table 6:** Parameter sensitivity of the proposed method in Scenarios C1 and C2.

Case	Parameter	Values	${\mathrm{RMSE}}_{p}$ (m)	${\mathrm{RMSE}}_{b}$ (°)	Min. $\Gamma_{t}$	$d_{\min}^{\mathrm{NFZ}}$ (m)
C1, proposed	$\kappa_{\beta }$	100/500/1000	16.209/16.208/16.208	1.901/1.901/1.901	3.324/3.437/3.437	21.046/21.304/21.304
	κ	$10^{4}/10^{5}/10^{6}$	22.615/16.208/20.097	1.724/1.901/2.032	3.966/3.437/2.871	37.330/21.304/16.081
	$\mu_{s}$	5/10/20	16.128/16.208/16.733	1.889/1.901/1.989	3.253/3.437/2.770	24.042/21.304/19.764
	$\eta_{\max}$	40/80/120	16.510/16.208/16.185	1.977/1.901/1.902	2.964/3.437/3.384	18.024/21.304/24.238
C2, proposed	$\kappa_{\beta }$	100/500/1000	16.413/16.413/16.413	1.741/1.741/1.741	4.976/4.976/4.976	29.256/29.256/29.256
	κ	$10^{4}/10^{5}/10^{6}$	16.704/16.413/16.240	1.763/1.741/1.736	4.565/4.976/4.605	30.374/29.256/20.048
	$\mu_{s}$	5/10/20	16.417/16.413/16.349	1.746/1.741/1.742	5.110/4.976/4.735	33.429/29.256/25.838
	$\eta_{\max}$	40/80/120	16.281/16.413/16.411	1.739/1.741/1.746	4.643/4.976/5.168	23.482/29.256/32.897

## Data Availability

The data are contained within the article.

## Abbreviations

The following abbreviations are used in this manuscript:

UAV	Unmanned aerial vehicle
NFZ	No-fly zone
FIM	Fisher information matrix
EKF	Extended Kalman filter
LOS	Line of sight
CRLB	Cramér–Rao lower bound
CBF	Control barrier function
QP	Quadratic programming
RMSE	Root mean square error

## Appendix A. Proof of Proposition 2

Let

$$C_{l}=\left\{x\in R^{2}:\gamma_{l}\left(x\right)\geq 1\right\}$$

and $h_{l}\left(x\right)=\gamma_{l}\left(x\right)-1$. To prove forward invariance of $C_{l}$, it is sufficient by Nagumo’s viability condition to show that

$${\dot{h}}_{l}\left(x\right)\geq 0,\;\forall x\in \partial C_{l}.$$

From the kinematics in Equation (1), the position dynamics are

$${\dot{p}}_{U}\left(t\right)=g\left(\psi \left(t\right)\right)v\left(t\right).$$

Taking the time derivative of $h_{l}$ gives

(A1) $${\dot{h}}_{l}\left(p_{U}\right)=\nabla_{x}\gamma_{l}{\left(p_{U}\right)}^{T}g\left(\psi \right)v.$$

Using the definition

$$a_{l}\left(x,\psi \right)=\nabla_{x}\gamma_{l}{\left(x\right)}^{T}g\left(\psi \right),$$

Equation (A1) becomes

(A2) $${\dot{h}}_{l}\left(p_{U}\right)=a_{l}\left(p_{U},\psi \right)v.$$

We now examine the sign of the normal velocity on $\partial C_{l}$. On this boundary,

$$h_{l}\left(p_{U}\right)=0.$$

If $a_{l}\left(p_{U},\psi \right)\geq 0$, then the current heading has no inward normal component. Since v≥0, Equation (A2) gives

$${\dot{h}}_{l}\left(p_{U}\right)=a_{l}\left(p_{U},\psi \right)v\geq 0.$$

If $a_{l}\left(p_{U},\psi \right)<0$, then the current heading points toward the unsafe side of the inner safety manifold. In this case, the speed bound in Equation (65) gives, on $\partial C_{l}$,

$$v_{l}\left(p_{U},\psi \right)=\frac{k_{l}h_{l}\left(p_{U}\right)}{-a_{l}\left(p_{U},\psi \right)}=0.$$

Therefore, from Equation (66),

$$v=\min\{v_{\mathrm{nom}},v_{l}\}=0.$$

Substituting this into Equation (A2) gives

$${\dot{h}}_{l}\left(p_{U}\right)=0.$$

Therefore, in both cases,

(A3) $${\dot{h}}_{l}\left(p_{U}\right)\geq 0,\;p_{U}\in \partial C_{l}.$$

Thus, on the inner safety manifold $\gamma_{l}\left(x\right)=1$, the UAV velocity does not generate any normal crossing component pointing into the set $\left\{x:\gamma_{l}\left(x\right)<1\right\}$. The boundary condition in Equation (A3) satisfies Nagumo’s viability condition for the continuous-time kinematics. Therefore, any trajectory satisfying

$$\gamma_{l}\left(p_{U}\left(0\right)\right)\geq 1$$

does not enter the region

$$\{x:\gamma_{l}\left(x\right)<1\}.$$

Therefore, the vector field points into or tangent to $C_{l}$ on $\partial C_{l}$. By Nagumo’s viability condition, $C_{l}$ is forward invariant. Hence, if $p_{U}\left(0\right)\in C_{l}$, then $p_{U}\left(t\right)\in C_{l}$ for all t≥0. This completes the proof.
